# Ultrasound localisation microscopy tracks testicular microvascular adaptations to endocrine function in male infertility

**DOI:** 10.1016/j.ebiom.2026.106333

**Published:** 2026-06-18

**Authors:** Nikoleta Papanikolaou, Jipeng Yan, Biao Huang, Rhianna Davies, Cecilia Dunsterville, Nipun Laksitha De Silva, Cara Go, Pasha Grachev, Rong Luo, Jacob Broughton-Venner, Suks Minhas, Gavin Bewick, Waljit Dhillo, Kevin Murphy, Adrian Lim, Meng-Xing Tang, Channa N. Jayasena

**Affiliations:** a Division of Diabetes, Endocrinology and Metabolism, Section of Endocrinology and Investigative Medicine, Imperial College London, London, UK; b Department of Bioengineering, Imperial College London, London, UK; c State Key Laboratory of Robotics and System, Harbin Institute of Technology, Harbin, China; d Department of Clinical Sciences, Faculty of Medicine, General Sir John Kotelawala Defence University, Ratmalana, Sri Lanka; e Department of Urology, Charing Cross Hospital, London, UK; f Department of Diabetes and Obesity, King's College London, London, UK; g Department of Imaging, Imperial College London and Healthcare NHS Trust, Charing Cross Hospital Campus, London, UK

**Keywords:** Testes, Super-resolution ultrasonography (SRUS), Ultrasound localisation microscopy (ULM), Male hypogonadism, Male infertility

## Abstract

**Background:**

Testicular disorders, including male infertility and hypogonadism, are increasingly prevalent and current diagnostic tools have important limitations. The testicular microcirculation underpins testicular function. Ultrasound localisation microscopy (ULM) enables super-resolution mapping of microvascular structure and flow at clinically relevant organ depth.

**Methods:**

Prospective case–control study of ULM-assessed testicular activity in men and rodents using clinical and research ultrasound systems. Study 1 compared healthy men (n = 10) with hypogonadotrophic hypogonadism (HH) (n = 9). Study 2 included men with HH receiving testosterone (n = 11), gonadotrophins (n = 9), or no treatment (n = 12). Study 3 assessed 12-month fertility treatment response in HH (n = 7). A rodent pubertal-blockade model was also studied (n = 5).

**Findings:**

ULM markers discriminated HH from controls (vessel density p < 0.01; diameter p = 0.01; tortuosity p < 0.01) and correlated with testosterone (r = 0.53–0.67, p < 0.05) and inhibin B (r = −0.61, p < 0.01). Vessel density, diameter, area and flow-related index were reduced in azoospermia (p < 0.01). ULM distinguished HH treatment groups (vessel density p < 0.001; diameter p < 0.05), with density and diameter correlating with testosterone (r = 0.69, 0.62; p < 0.001) and inhibin B (r = 0.64, 0.65; p < 0.001). Vessel density (p < 0.001) and diameter (p < 0.01) were reduced in azoospermia irrespective of treatment. During fertility therapy, ULM parameters increased (p < 0.05) and detected testicular activation earlier than volume or inhibin B. In rodents, pubertal development showed dynamic microvascular remodelling driven by testis growth.

**Interpretation:**

ULM provides a treatment-responsive, biologically grounded biomarker of testicular function enabling patient stratification, early detection of therapeutic response, and potential for both refinement of clinical decision-making in HH, and application within other testicular disorders.

**Funding:**

MRC, NIHR Biomedical Research Centre Funding Scheme and the NIHR/Imperial Clinical Research Facility, Diabetes UK, BBSRC, MRC, Imperial Private Healthcare Clinical Research Fellowship Scheme, NWLP Research Grant.

**Research in context**

**Evidence before this study**

Recent studies have established ULM as a novel imaging technique based on ultrasound. ULM enables visualisation of micro-circulation in experimental animal and human models including the brain, kidney, heart, and tumours. ULM allows quantification of microvascular structure and flow characteristics correlating with organ function, which contrasts with other imaging modalities such as classical ultrasound and MRI. The unprecedented resolution achievable by this technique in vivo offers a potentially powerful clinical tool without the need for invasive approaches.

The prevalence of male reproductive disorders such as male infertility has doubled since the 1970s. Current investigative tools such as semen analysis and serum reproductive hormone measurement have limitations. Innovations to improve diagnosis and eventually treatment of male reproductive disorders are needed.

**Added value of this study**

We show that ULM can be used successfully within a conventional ultrasound system, to track low-velocity microvascular blood flow within the testes. We use a classical, reversible model of male infertility (hypogonadotrophic hypogonadism) to show that ULM is a highly sensitive tool for assessing testicular function. Men with HH have reduced microvasculature compared with healthy controls, and ULM markers had better discriminatory capacity compared with clinical markers. ULM-markers correlated with canonical biochemical and spermatogenic markers of testicular function, implicating the microvasculature as a novel means of assess testicular function. Additionally, pharmacological reversal of HH over a 12-month period was detected more rapidly with ULM compared with clinical and biochemical markers in affected men. Our data implicate ULM as an image processing tool that could be incorporated within clinical scanners to improve the assessment of testicular function in men.

**Implications of all the available evidence**

ULM could become a powerful tool to provide further insight into the microvasculature-function association. This work sets the foundation for ULM utilisation in the field of male reproductive health and paves the way for future studies of greater scale, inclusive of other causes of male infertility, and studies upon optimising surgical sperm retrieval.

## Introduction

The testes are essential for puberty, sexual function, and fertility in men.^1^ Disorders of testicular function, including infertility and hypogonadism, are increasingly common due to rising obesity, diabetes, and age-related co-morbidities, and severely impact quality of life in affected men and couples.^2^^,^^3^ A 2017 systematic review reported a fall in total sperm counts by 59.3% since the 1970s, in North America, Europe, and Australasia.^4^ Subsequently, a recent meta-analysis also demonstrated a decline in sperm count in men from South/Central America-Asia-Africa, making this a global decline.^5^ Additionally, data suggest a progressive increase in risk of testicular germ cell cancer, a probable increase in risk of cryptorchidism and hypospadias pointing towards environmental/lifestyle causes.^6^ Men with disorders of testicular function have increased risks of heart disease, insulin resistance, anaemia, osteoporosis, depression, and testicular cancer.^7^ Clinically available tests of testicular function have changed little in 20 years. Semen analysis directly assesses fertility but requires ejaculation, which can be embarrassing or unacceptable.^8^ Circulating testosterone measurement is limited by diurnal variation, food suppression, poor assay harmonisation, and overlap between hypogonadal and eugonadal men.^1^ Novel technologies to identify reproductive disorders are therefore needed.

Testicular ultrasonography (US) is an effective and widely accessible modality for evaluating potential testicular/peri testicular abnormalities that could impact testicular function and fertility potential such as cryptorchidism and varicoceles. US is considered the gold standard for the measurement of testicular volume, and several studies have proven a weak to moderate volume-function association.^9^, ^10^, ^11^ Additionally, retrospective studies demonstrated a higher prevalence of US abnormalities (such as testicular hypotrophy and inhomogeneity) as well as differences in doppler-derived parameters (such as resistive index of the testicular artery) in men with impaired semen parameters compared with controls.^12^, ^13^, ^14^, ^15^ However, information obtained from brightness (B) or Doppler mode do not reliably serve as informative markers of testicular function and therefore the role of current imaging modalities in evaluation of male infertility is debated in international guidelines.^16^ Magnetic Resonance (MR) imaging is usually employed in clinical practice when US fails to provide conclusive findings on testicular lesions or when detailed anatomy of the reproductive tract is needed.^17^ Contrast-enhanced ultrasound (CEUS) with gas-filled microbubbles (MB) enhances vascular imaging. CEUS-measured testicular blood flow has been measured in men with Klinefelter’s syndrome (primary hypogonadism), and correlated with altered endocrine function.^18^ However, CEUS has diffraction-limited resolution. Furthermore, quantitative CEUS metrics such as acoustic intensity or time–intensity curves are affected by probe position, MB distribution, and scanner settings, which make it better suited for qualitative purposes.^19^

Ultrasound localisation microscopy (ULM), a super-resolution ultrasonography (SRUS) method, overcomes the diffraction limit by localising MB with sub-pixel precision.^20^^,^^21^ Tracking isolated MB across frames enables visualisation of microvascular circulation, including flow velocity and direction, at unprecedented resolution.^22^^,^^23^ ULM achieves tens-of-microns resolution deep within tissues, bridging imaging and histopathology. It shows promise in diverse clinical applications, including tumour microvascular pathology, brain vascular abnormalities, and myocardial small-vessel disease.^24^, ^25^, ^26^, ^27^, ^28^ ULM of testes linked testicular microcirculation to spermatogenic function in men with non-obstructive azoospermia (NOA) and obstructive azoospermia.^29^

The microcirculation adapts to tissue metabolic needs^30^^,^^31^ and has a critical role in testicular function namely spermatogenesis, hormonal and paracrine control in mammalian testis.^32^ Sperm quality and quantity depend on tissue perfusion within the testis^33^ and testicular blood flow positively correlates with serum testosterone levels.^34^ Sex hormones modulate the testicular microvasculature with regards to blood flow, permeability and vasomotion which subsequently regulates Leydig cells function and gametogenesis.^32^^,^^35^^,^^36^ Therefore, we hypothesised that ULM could sensitively measure testicular function. To test our hypothesis, we recruited men with hypogonadotrophic hypogonadism (HH) as a model for men with reduced testicular function. Male HH is characterised by impairment of the hypothalamus and/or pituitary gland affecting the secretion of gonadotrophins, follicle-stimulating hormone (FSH) and luteinising hormone (LH), resulting in insufficient testicular function and deficiencies in testosterone and spermatogenesis. Testosterone replacement therapy (TRT) is the cornerstone of treatment of male hypogonadism, but it does not restore spermatogenesis. HH is one of the few medically treatable causes of male infertility with exogenous pulsatile gonadotrophin-releasing hormone (GnRH) and gonadotrophins restoring normal serum testosterone/intratesticular testosterone and inducing spermatogenesis.^37^

Herein, in proof-of-concept clinical studies using a commercial ultrasound device, we showed ULM could map human testicular microvasculature at 90 μm resolution, beyond the diffraction limit, and that ULM-derived vascular parameters correlated with biochemical and spermatogenic markers in men with HH. We then applied a high-frame-rate research scanner in rodents to relate ULM-derived microvascular changes to histological and transcriptomic markers of testicular function under pharmacologically induced inactivation.

## Methods

### Human studies

#### Ethics

The study was approved by the London–West London & Gene Therapy Advisory Research Ethics Committee (REC 21/PR/0717) and was conducted in accordance with the principles of the Declaration of Helsinki. All participants gave informed consent.

#### Participant recruitment

Healthy men (18–60 years) with normal reproductive history, sex hormones, and semen analysis were recruited using paper, online adverts, and the volunteer database of Imperial Clinical Research Facility (ICRF). Men with HH (18–60 years) had testosterone <8 nmol/L, free testosterone <220 pmol/L, and gonadotrophins either in the normal or below the reference range. Men with HH were recruited from the endocrinology and reproductive clinics within Imperial College Healthcare Trust (ICHT). Both congenital and acquired forms of HH were included. The recruitment period took place from November 2021–May 2023. The study visits were conducted in ICRF, Hammersmith Hospital campus and in the Radiology department, ICHT. Exclusion criteria were systemic co-morbidities, opioid/androgen abuse, testicular surgery, cryptorchidism, > ten pack-years smoking, acute illness, or contraindications to microbubble contrast agent.

#### Study 1: HH versus controls

Healthy men (n = 10) and HH men (n = 9) each underwent one CEUS–ULM, serum hormones, and semen analysis.

#### Study 2: HH after therapy

HH men were grouped as TRT (n = 11), ≥12 months gonadotrophins (n = 9), or treatment naïve (n = 12). Each underwent CEUS–ULM, serum hormones, and semen analysis.

#### Study 3: longitudinal therapy response

HH men commencing gonadotrophins for fertility (n = 7) were studied at 0 (baseline, prior to gonadotrophins commencement), 3, 6, and 12 months. Treatment followed Society for Endocrinology guidelines.^38^ In brief, pre-pubertal HH received human chorionic gonadotrophin (hCG) + menotrophin. Post-pubertal HH began hCG, with menotrophin added if azoospermic at 3 months. HCG was titrated (1, 3, 6 months) to mid-range testosterone; menotrophin adjusted by FSH/sperm. HH men on established TRT (n = 7) were studied at the same timepoints. At each visit, CEUS–ULM and blood tests were performed; semen analysis was limited to the gonadotrophin group.

### Hormone measurement

Fasting morning blood (08:00–11:00) was collected for serum testosterone, sex hormone-binding globulin (SHBG), LH, FSH, oestradiol (E2), and inhibin B. Analyses were performed in the Clinical Biochemistry Department, Charing Cross Hospital, on the ARCHITECT platform (Abbott, Chicago, IL, USA) under UK National External Quality Accreditation Service (UKNEQAS) accreditation. Male reference ranges: LH 2–12 IU/L; FSH 1.7–8 IU/L; testosterone 10–30 nmol/L; SHBG 15–55 nmol/L; inhibin B 25–325 ng/L.

### Semen analysis

Samples were obtained after 2–7 days abstinence and analysed within 60 min as per WHO 6th Edition guidelines^39^ in the andrology laboratory of ICHT under UKNEQAS accreditation. Parameters included semen volume, sperm concentration, total count, progressive motility, total motility, and morphology.

### Rodent study

#### Ethics

The study was conducted at Imperial College Centre for Biomedical Services under Home Office licence PD75F462C and followed ARRIVE guidelines. All animal studies were conducted according to the Animals (Scientific Procedures) Act 1986 Amendment Regulations 2012 and approved by the Animal Welfare Ethical Review Body at Imperial College London.

#### Study 4: pubertal blockade in rats

Pre-pubertal male Wistar rats (n = 10; 70–90 g upon arrival; Charles River, Margate, UK) were group housed in individually ventilated cages under controlled conditions (21–23 °C; 12:12 h light-dark cycle, lights on at 7:30 a.m.). The rats at postnatal day (pnd) 24 were randomised to treatment with either GnRH antagonist (Degarelix) to block puberty or vehicle control (0.9% NaCL),^40^ (n = 5/group). Baseline testis imaging was at pnd 21; repeat imaging at pnd 56, followed by sacrifice. The rats were handled daily and weighed on the first day of the experiment, followed by three times a week during the experiment. The rats had ad libitum access to standard chow.

#### Histology

The left testis (arbitrary selection) was excised and sent for histopathological analysis. Twenty perforations were made to the visceral lamina to allow the fixing solution into the tissue. The testes were placed in Bouin’s solution at 4 °C overnight. The next day, the samples were rinsed and placed in 70% ethanol and kept at 4 °C. The tissue processing (fixation, dehydration, clearing, wax infiltration) was performed using a tissue processor. The prepared tissues were then embedded in paraffin, and sections of tissue were cut longitudinally using a microtome. Haematoxylin and eosin staining was used for microscopic analysis.

#### RNA extraction and sequencing

The contralateral testes were used for this procedure. Testis samples were homogenised from frozen for three 1-min cycles in 1 ml of TRIzol Reagent, (REF15596026; Thermo Fisher Scientific, Darford, UK) using a TissueLyser II (Qiagen, Hilden, Germany) homogeniser. The samples were incubated on ice for 1 min between cycles. Chloroform (200 μl; REF22711.324; VWR International, Lutterworth, UK) was added to each sample, before being spun at 10,000 rpm for 10 min at 4 °C. The upper phase of the centrifuged sample was collected and precipitated using 0.5 ml of 70% isopropanol (CAS 67-63-0; Sigma Aldrich, St. Louis, MI, USA). The samples were spun at 12,500 rpm for 15 min, and the upper phase was discarded. The pellets were washed in 70% ethanol (CAS 64-17-5; Sigma Aldrich) and left to dry, before being resuspended in RNAse free water (REF AM9932; Thermo Fisher Scientific). RNA sequencing was done by Novogene (Cambridge,UK). In brief, Messenger RNA was purified from total RNA using poly-T oligo-attached magnetic beads. After fragmentation, the first strand cDNA was synthesised using random hexamer primers followed by the second strand cDNA synthesis. The library was ready after end repair, A-tailing, adapter ligation, size selection, amplification, and purification. The library was checked with Qubit and real-time PCR for quantification and bioanalyser for size distribution detection. Quantified libraries will be pooled and sequenced on Illumina platforms, according to effective library concentration and data amount.

#### Pathway analysis

The RNAseq dataset (n = 10) was submitted for Ingenuity Pathway Analysis (http://www.ingenuity.com/). A core analysis was conducted using a cutoff p value of <0.05 and a log2fold change < −3 and >3; this reduced the analysis ready dataset to 7184 genes. Benjamini-Hochberg correction (p < 0.05, z > 2 for -score) was applied for multiple comparisons.

### Imaging protocol

#### Human studies

CEUS was performed using Aplio i800 ultrasound machine (Canon Medical Systems, Otawara, Japan) with an ultra-wideband Linear i18LX5 transducer (Canon Medical Systems; imaging frequency: 6 MHz, mechanical index (MI: 0.03) of choice with a frame rate of 13 Hz. Participants were invited to lay supine on an examination couch. Initially, dimensions of each testis, volume measurements and echotexture characterisation were obtained at B mode. Testicular volume was automatically calculated based on the formula: length × width × anteroposterior diameter) × π/6. During CEUS imaging, participants were asked to minimise any movement whilst the radiologist stabilised their hand holding the probe. The contrast agents were sulphur hexafluoride (Sonovue®, Bracco, Milan) microbubbles dosed at 2.4 mL for imaging each testis. This was given by a single study investigator as a slow intravenous injection (approximately 15 s) followed by a 5 ml normal saline as a bolus flush based on a timer on the screen and in accordance with that. The dose of contrast and the rate of injection was decided in a standardisation and optimisation phase prior to study commencement to allow optimal localisation and tracking of MBs (results not shown). Both testes were scanned in the maximum diameter-at two planes: Longitudinal scan (Sagittal plane of testis)–ensuring maximal length of testis so maximum view; Orthogonal transverse scan (axial plane of testis)–ensuring true orthogonal plane using mediastinum testis as a marker and maximum transverse diameter of testis in view. The planes selected are standard of care views and were chosen as they could be reproduced and standardised for each participant. Data were acquired in the form of 1-min videoclip following contrast administration. The B-mode videos and CEUS videos were recorded simultaneously and saved in the clinical US machine. Total four video clips per participant (two planes per testis) were collected for ULM analysis providing a total of 3120 frames per participant.

#### Rodent study

Anaesthesia was induced and maintained via inhalation anaesthesia (Isoflurane; Zoteris, Leatherhead, UK) and maintained at 1.5%–2% in O2 delivered via a nose cone at 2 L/min. The animals were placed on a heat pad and eye gel was applied to protect their eyes. Clippers followed by depilation cream (Veet, UK) were used to remove the fur from the left testis. The rodents were placed in a supine position and their left testis (arbitrary selection) was scanned using a high frequency ultrasound probe (L22-14Vx; Verasonics, Kirkland, WA, USA; imaging frequency: 18 MHz, MI 0.16), connected to a Vantage 256 system (Verasonics) via a 128-channel interface (UTA 260-D; Verasonics). The MI was measured in the lab using a hydrophone (SN2344, Precision Acoustics). The testis was imaged at two planes similarly to the human protocol. The imaging pulse repeat frequency was 3593 Hz, and 11 steering angles were used for coherent angle compounding (−11 to 11°). The final imaging frame rate was 250 Hz. The contrast agent used was a homemade MB solution used in previous experiments.^41^ In brief, the contrast was made of a lipid colloid solution of 1 mg/ml in a 2 ml glass vial to which gaseous perfluorobutane (F2 Chemicals, Lea, UK) was added. The vial was shaken for 1 min in an amalgamator and the MB solution was diluted 1:14 in saline. The rodents received 1 ml/kg of the MB-saline dilution intravenously via a tail vein catheter 30 min after the induction of anaesthesia. Keeping this timing consistent is important as it standardises the vasodilatory effect of isoflurane across animals and imaging sessions.^42^ The animals were anaesthetised for a total of 40 min. Four 1-s videos (1920 frames) from each plane (longitudinal and transverse) were reconstructed and concatenated for later processing. Contrary to the clinical ultrasound machine where the reconstructed videos were exported directly, the radiofrequency (RF) signals were saved for later reconstruction and processing. Thereafter, the B-mode images were formed by using the conventional delay-and-sum algorithm. CEUS videos were formed by using the singular value decomposition (SVD)-based spatial and temporal filtering on the RF signal.^43^^,^^44^ The SVD cutoff was empirically set as first 15 singular vectors.

### ULM analysis

For the human studies, ULM analysis was performed on exported DICOM videos. The DICOM videos were cropped to remove any US related labels/annotations. The motion from respiration, cardiac cycle, cremaster reflex and hand motion was required to be compensated for in order to achieve the high-resolution images.^45^ The tissue motion was modelled as an affine transformation and was estimated for each frame in the B mode video by an optimization method to minimise the difference from the reference frame, which was set as the first frame in the B mode video. In the rodent study, non-rigid motion compensation was added, considering the heavier motion from the higher heart rate and respiration. The estimated motion field was then applied on the CEUS videos to compensate for the MB motion from the tissue. The localisation of MB was performed on each CEUS frame. Noise in MB images was reduced by an empirically set global background noise threshold. The point spread function (PSF) was estimated via a gaussian fitting on ten manually segmented MB patches. Normalised cross-correlation coefficient map was calculated between each MB image and PSF. The MB were then localised from the local maxima of regions on the correlation coefficient map where the coefficients were set above 0.5.

The location of MB on each frame were then fed into a Kalman-based MB tracking framework^46^ to allow MB pairing between frames and to estimate the MB flow speed and direction.^46^ An empirical speed threshold of 5 mm/s was set for the human dataset because the low frame rate impaired tracking of faster MBs; for the rodent dataset, which had a higher frame rate, the threshold was increased to 25 mm/s. MB tracks that were less than 3 frames were filtered out, to reduce the erroneous tracks from the noise.

**Fig. 1:**
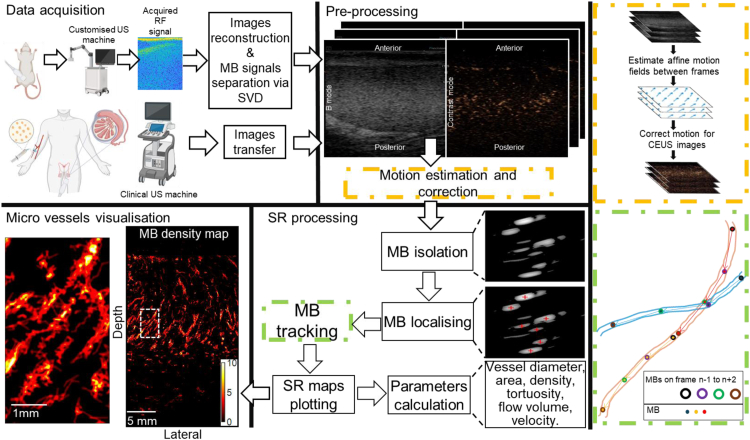
**Schematic of data acquisition and processing methods.** MB, microbubbles; CEUS, contrast enhanced ultrasound; SR, super resolution; SVD, singular value decomposition.

For qualitative visualisation, microvascular maps were then reconstructed via the interpolation and accumulation of MB trajectories. The flow dynamics can be described by flow speed and directional maps (Fig. 1). Microvascular quantitative parameters, namely vessel density, diameter, tortuosity, velocity, vessel area and flow related index (FRI) were calculated from the region of interest (ROI) on the super resolution maps. The ROI was manually selected by a blinded clinician and included the whole intratesticular area, avoiding the capsule by a few mm. Vessel density was defined as the ratio of number of pixels corresponding to vessels to number of pixels of whole ROI ($vessel\;density=\frac{\text{pixels}\;\text{in}\;\text{vessel}\;\text{area}}{\text{pixels}\;\text{in}\;\text{whole}\;\text{ROI}}$). Tortuosity (t) refers to the ratio of the total length of each track (C) to the direct distance between its ends (L), i.e., t = C/L. Velocity was measured in mm/sec. Vessel diameter was calculated by firstly extracting the vessel regions by binarizing super-resolution MB density map with an empirical threshold, i.e., 0.9 of the peak intensity of a single MB trajectory. The centreline of the vessels was then extracted, and the diameter was calculated as double the distance between the centreline and its nearest vessel boundary. Diameter was measured in μm. Vessel area refers to the total area within the ROI occupied by the extracted vascular region and is measured in mm^2^. FRI represents a flow-related quantitative metric, rather than a true volumetric measure and is reported in arbitrary units (a.u). FRI is calculated by summing up MB trajectories within the ROI. Under the assumption of constant MB concentration, the accumulated MB trajectories within a standardised imaging plane over a fixed acquisition duration are expected to correlate with the amount of blood traversing the field of view. Accordingly, FRI is best interpreted as a relative indicator of blood flow, enabling comparative assessment of regional perfusion across subjects. Assuming that vascular distribution would have been the same in the two planes and by recruiting men without known lateralising testicular issues, we averaged the data from the two planes and subsequently from the two testes to form single parameters for each participant. The rodent study only included imaging of the left testes and similarly the data were averaged from the two planes.

To estimate the achieved resolution by the super resolution imaging, a Fourier ring correlation-based method was used. Similar to previous studies, the MB tracks were randomly split into two subsets, from which two trajectories’ images were then reconstructed.^47^^,^^48^ A Fourier ring correlation curve was then generated from the image pair, the cross between this curve and a ½ bit information curve was used for the estimation of resolution.

### Statistical analysis

#### Human studies

Our studies are exploratory and pilot in nature measuring multiple outcomes and look for patterns in the data and relationships. There are no prior studies of ULM for testicular function. We aimed to recruit 12 men per group based on recommendations when there is uncertainty about effect size.^49^ Due to motion artifacts rendering their CEUS unsuitable for analysis, the sample size in each group at the start of the study is different from the n numbers in the analysis. The latter is provided in the manuscript. Additionally, due to the small number of men with HH seeking fertility and eligibility for gonadotrophins therapy as per standard care and difficulty in retention for a year, the final n number for the analysis was lower for the 3rd study.

#### Rodent study

The ULM parameter vessel density was consistently altered in the human studies, and we therefore powered the rodent study to detect a change in vessel density. In humans with HH, vessel density was approximately 60% lower than controls. Based on a standard deviation from preliminary rodent studies, we calculated that to detect a change in vessel density of 50% with power of 80% at a level of significance of 0.05 we would need 4 animals per group. N = 5/group was chosen to account for attrition.

#### All studies

Data normality was assessed by Shapiro–Wilk and QQ plots. Normally distributed data are presented as mean ± SD; non-normal as median [percentiles]. Categorical data are presented as frequencies of counts. Degree of association between two variables was estimated using Pearson’s correlation coefficient or Spearman’s rank correlation coefficient where associations were suspected to be non-linear. Group comparisons used student’s two tailed t-test or Mann–Whitney U for continuous unpaired data (or paired t-test/Wilcoxon test for paired data), Chi-square/Fisher for categorical data. Receiver operating characteristic (ROC) curves were generated, with an area under the curve (AUC) of 0.5 and 1 denoting non- and total discrimination, respectively, and an AUC greater than 0.9 typically considered as indicating an outstanding diagnostic indicator. Study 2 treatment groups were compared by ANOVA with Bonferroni correction. Homogeneity of variance was assessed by Brown-Forsythe test. Non-normal data were log-transformed or analysed with Kruskal–Wallis. Study 3 longitudinal data were analysed by two-way ANOVA (or mixed-effects model if missing values), including group × time interaction. Bonferroni’s multiple comparisons test was used post hoc to investigate differences between groups and timepoints. Data are presented graphically as time profiles of means and standard deviation. In all cases, p < 0.05 was considered statistically significant. Statistical analyses were performed using Prism statistical package (GraphPad Software Inc, San Diego, USA).

### Role of funders

The funders had no role in the study design, data collection, data analysis, interpretation, or report writing.

## Results

### Study 1: ULM parameters in men with hypogonadotrophic hypogonadism (HH) versus controls

Ten healthy men and nine with HH were recruited. Flow diagram of the study participants can be found in Supplementary Fig. S1. Age and BMI were similar between groups (Table 1). As expected, testicular volume (7.5 [6.8–10.0] versus 14.9 [10.8–18.8] mL, p = 0.01, by Mann–Whitney U test), and serum testosterone (3.8 ± 3.0 versus 16.0 ± 4.0 nmol/L, p < 0.001, by student’s two tailed t-test), LH (0.8 ± 0.5 versus 2.4 ± 0.7 IU/L, p < 0.001, by student’s two tailed t-test), FSH (1.5 ± 1.0 versus 2.9 ± 1.3 IU/L, p < 0.05, by student’s two tailed t-test) and inhibin B (130.0 ± 82.8 versus 209.0 ± 63.6 ng/L, p < 0.05, by student’s two tailed t-test) were reduced in men with HH compared with controls. All HH men had impaired semen quality (6/9 azoospermic), while all controls had normal semen parameters (Table 1). Additional demographic and anthropometric data can be found in Supplementary Table S1.

**Table 1:** Characteristics of men with hypogonadotrophic hypogonadism versus controls.

Baseline characteristic (units)	HH group (n = 9)	Control group (n = 10)	p value
Age (years)	47.0 ± 10.6 (38.9–55.1)	38.4 ± 9.6 (31.5–45.3)	0.08
BMI (kg/m^2^)	26.9 ± 2.3 (25.2–28.7)	25.5 ± 3.4 (23.1–27.9)	0.30
Testicular volume (ml)-median [percentiles]	7.5 [6.8, 10] (6.4–11.3)	14.9 [10.8, 18.8] (9.7–19.9)	0.01^c^
Hormonal profile			
Testosterone (nmol/L)	3.8 ± 3.0 (1.5–6.1)	16.0 ± 4.0 (13.2–18.9)	<0.0001^c^
FSH (IU/L)	1.5 ± 1.0^a^ (0.6–2.4)	2.9 ± 1.3 (2.0–3.8)	0.03^c^
LH (IU/L)	0.8 ± 0.5^a^ (0.4–1.2)	2.4 ± 0.7 (2.0–2.9)	<0.0001^c^
Inhibin B (ng/L)	130.0 ± 82.8^a^ (61.0–199.0)	209.0 ± 63.6 (163.0–254.0)	0.04^c^
Semen parameters			
Semen volume (ml)	3.0 ± 1.0^b^ (2.1–3.8)	3.8 ± 2.1 (2.2–5.3)	0.35
Sperm concentration (millions/ml)-median [percentiles]	0.0 [0.0, 0.96]^b^ (0.0–80.0)	41.7 [18.1, 75.1] (17.6–80.0)	0.0035^c^
Azoospermia	6	0	<0.001^c^
Sperm seen	2	10	

Continuous data are presented as mean ± standard deviation (95% CI) if normally distributed and median [IQR] (95% CI) if non normally distributed. Categorical data are presented as frequencies of counts.

HH, hypogonadotrophic hypogonadism; BMI, body mass index; FSH, follicle-stimulating hormone; LH, luteinising hormone.

a Contains missing data due to non-available results of a single participant, (n = 8).

b Contains missing data due to another participant’s refusal to have a semen analysis, (n = 8).

c Denotes where differences between groups were statistically significant at p < 0.05 by Student’s two-tailed t-test for parametric, Mann-Whitney U test for non-parametric data, Chi-Square test for categorical data.

**Fig. 2:**
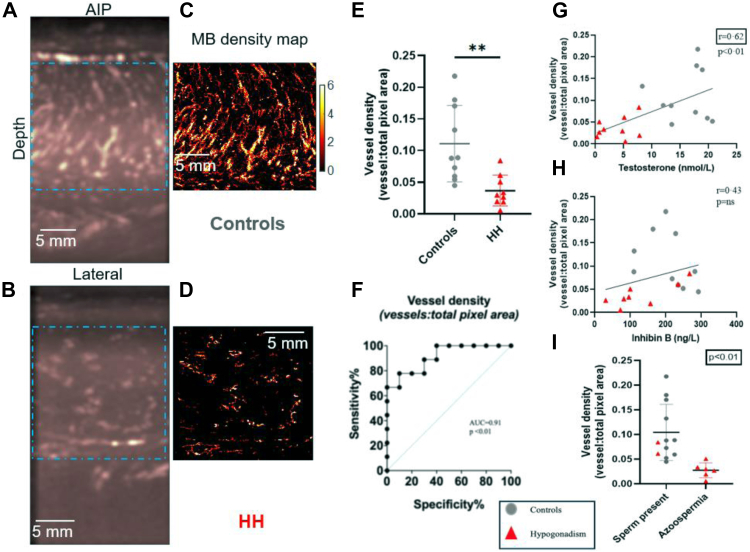
**Men with hypogonadotrophic hypogonadism have lower density of the testicular microvasculature compared with healthy men.** (A, B): Examples from temporal average intensity project of recorded CEUS images in the longitudinal plane from control group and HH group. (C, D) Super resolution image from dashed area in A and B demonstrating super-localised MB in controls and in men with HH; (E): Column scatter plot of vessel density as calculated with super resolution image processing in the intratesticular area in controls and men with HH. Data are displayed as mean ± standard deviation. ∗∗p < 0.01, by Student’s two-tailed t-test. N = 9 (men with HH) and N = 10 (healthy men); (F): Receiver operating characteristic curve for vessel density. (G): Scatter plot demonstrating correlation between vessel density and serum testosterone by Spearman’s correlation. N = 9 (men with HH) and N = 10 (healthy men); (H): Scatter plot demonstrating correlation between vessel density and inhibin B, by Spearman’s correlation. N = 8 (men with HH) and N = 10 (healthy men) (I): Column scatter plots comparing vessel density in men with sperm versus azoospermia, by Student’s two-tailed t-test. Data are displayed as mean ± standard deviation. N = 8 (men with HH) and N = 10 (healthy men). MB, microbubbles; HH, hypogonadotrophic hypogonadism. The super resolution images from the transverse plane can be seen in Supplementary Fig. S17.

ULM showed reduced vessel density (0.04 ± 0.02 versus 0.11 ± 0.06, p < 0.01, by student’s two tailed t-test), diameter (71 ± 11 μm versus 88 ± 15 μm, p = 0.01, by student’s two tailed t-test), area (24 ± 24 mm^2^ versus 72 ± 36 mm^2^, p < 0.01, by student’s two tailed t-test), and FRI (0.81 ± 1.07 versus 2.38 ± 1.39, p < 0.01, by student’s two tailed t-test) in HH (Fig. 2A–E and Supplementary Fig. S2); velocity did not differ (Supplementary Fig. S2). No extra-testicular differences were observed (data not shown). ROC curves demonstrated strong discrimination: vessel density AUC 0.91; diameter 0.88; area 0.90; FRI 0.90 (all p < 0.01, by ROC curve) (Fig. 2F and Supplementary Fig. S2).

ULM showed increased vessel density, diameter, area, and FRI in men with detectable sperm compared with men who had azoospermia (Fig. 2I and Supplementary Fig. S3). These ULM characteristics correlated with testosterone level (r = 0.53–0.67, p < 0.05, by Pearson’s or Spearman’s correlation (Fig. 2G, Supplementary Fig. S4); but not with inhibin B (Fig. 2H, Supplementary Fig. S5).

**Fig. 3:**
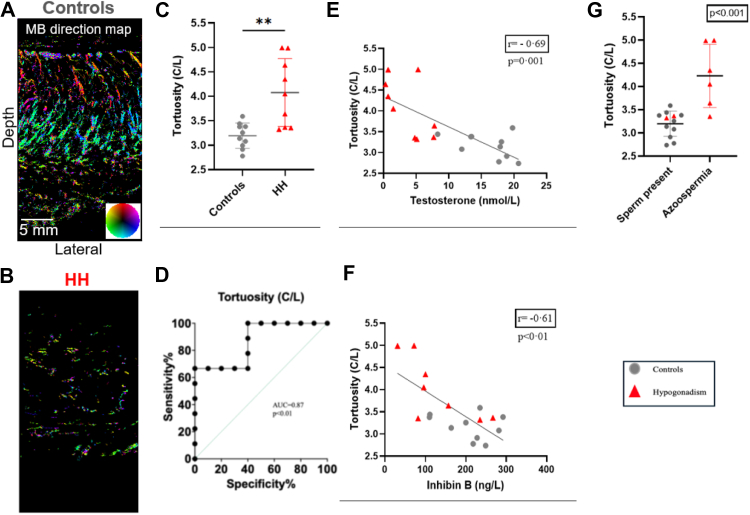
**Men with hypogonadotrophic hypogonadism have higher tortuosity of the testicular microvasculature compared with healthy men.** (A): Super resolution angle maps in controls; (B): Super resolution angle maps in men with HH; (C): Column scatter plot of tortuosity as calculated with super resolution image processing in the intratesticular area in controls and men with hypogonadotrophic hypogonadism. Data are displayed as mean ± standard deviation. ∗∗p < 0.01, by Student’s two-tailed t-test. N = 9 (men with HH) and N = 10 (healthy men); (D): Receiver operating characteristic curve for tortuosity; (E): Scatter plot demonstrating correlation between tortuosity and serum testosterone, by Spearman’s correlation. N = 9 (men with HH) and N = 10 (healthy men); (F): Scatter plot demonstrating correlation between tortuosity and inhibin B, by Spearman’s correlation. N = 8 (men with HH) and N = 10 (healthy men); (G): Column scatter plots comparing tortuosity in men with sperm versus azoospermia, by Student’s two-tailed t-test. N = 8 (men with HH) and N = 10 (healthy men). MB, microbubbles; HH, hypogonadotrophic hypogonadism.

Directional ULM suggested greater tortuosity in HH. Automated analysis confirmed this (4.1 ± 0.7 versus 3.2 ± 0.3, p < 0.01; AUC 0.88, by ROC curve (Fig. 3A–D). Tortuosity correlated negatively with testosterone (r = −0.69, p = 0.001, by Spearman’s correlation) (Fig. 3E) and inhibin B (r = −0.61, p < 0.01, by Spearman’s correlation) (Fig. 3F). Men with azoospermia had higher tortuosity compared with men who had sperm (p < 0.001, by student’s two tailed t-test) (Fig. 3G).

MB tracking results for Fig. 2A and B can be found in Supplementary Videos S1 and S2.

### Study 2: ULM in men with HH after gonadotrophin versus testosterone treatment

Gonadotrophins, but not TRT, reverse the inhibitory effects of HH on testicular function. HH men were grouped as untreated (n = 12), TRT (n = 11), or gonadotrophin-treated (n = 9; median duration of treatment 17 months). Flow diagram of the study participants can be found in Supplementary Fig. S6. Age, aetiology, and pre-treatment parameters were similar (Table 2 and Supplementary Table S2). Serum testosterone rose with TRT and gonadotrophins. Sperm was more frequent with gonadotrophins (7/9) versus untreated (3/12) or TRT (2/10, p < 0.05, by Fisher’s exact test). Established surrogate markers of spermatogenesis, testicular volume and inhibin B levels were non-significantly higher in the gonadotrophin group when compared with the other groups (Table 2). Additional demographic and anthropometric data can be found in Supplementary Table S2.

**Table 2:** Characteristics of men with hypogonadotrophic hypogonadism following gonadotrophin versus testosterone treatment.

Baseline characteristic (units)	HH on gonadotrophins n = 9	HH off-treatment n = 12	HH on TRT n = 11	p value
Age (years)	37.2 ± 4.3 (33.9–40.6)	37.8 ± 5.2 (34.6–41.1)	42.8 ± 10.8 (35.5–50.1)	0.18
BMI (kg/m^2^)	26.6 ± 4.5 (23.2–30.0)	31.4 ± 6.8 (27.1–35.7)	28.2 ± 6 (24.1–32.2)	0.18
Mean testicular volume (ml)	8.8 ± 5.8 (4.3–13.3)	6.5 ± 5.7 (2.8–10.1)	7.3 ± 3.6 (4.9-9.7)	0.58
Hormonal profile				
Testosterone (nmol/L)	18.5 ± 8.5 (12.0–25.1)	2.8 ± 2.1 (1.5–4.1)	16.7 ± 4 (14.0–19.4)	<0.0001^b^^c^
Oestradiol (pmol/L)				<0.001^b^
<100	2	12	5	
>100	7	0	6	
Inhibin B (ng/L)-median [percentiles]	79 [49, 106] (45–111)	40 [22,131] (20–145)	52 [30, 96] (22–99)	0.77
Semen parameters				
Volume (ml)- median [percentiles]	2.6 [1.8, 3.7] (1.6–4.3)	1.7 [1, 3.4] (1.0–3.7)	3.3 [2.5, 4.6]^a^ (2.4-4.7)	0.09
Sperm Concentration (million/ml)—median [percentiles]	6.4 [1.8, 25] (0.0–37.0)	0 [0, 1.7] (0.0–2.3)	0 [0, 0.03]^a^ (0.0-0.1)	0.009^b^^d^
Sperm concentration			a	0.02^b^
Azoospermia	2	9	8	
Sperm seen	7	3	2	

Continuous data are presented as mean ± standard (95% CI) deviation if normally distributed and median [IQR] (95% CI) if non normally distributed. Categorical data are presented as frequencies of counts.

HH, hypogonadotrophic hypogonadism; BMI, body mass index; FSH, follicle-stimulating hormone; LH, luteinising hormone; TRT, testosterone replacement therapy.

a Contains missing data, (n = 10).

b Denotes where differences between groups were statistically significant at p < 0.05, by one-way ANOVA for continuous data or Fisher’s exact test for categorical data.

c HH off treatment versus HH on gonadotrophins (p < 0.001) and HH off treatment versus HH on TRT (p < 0.001).

d HH off treatment versus HH on gonadotrophins (p = 0.04) and HH on TRT versus HH on gonadotrophins (p = 0.01).

**Fig. 4:**
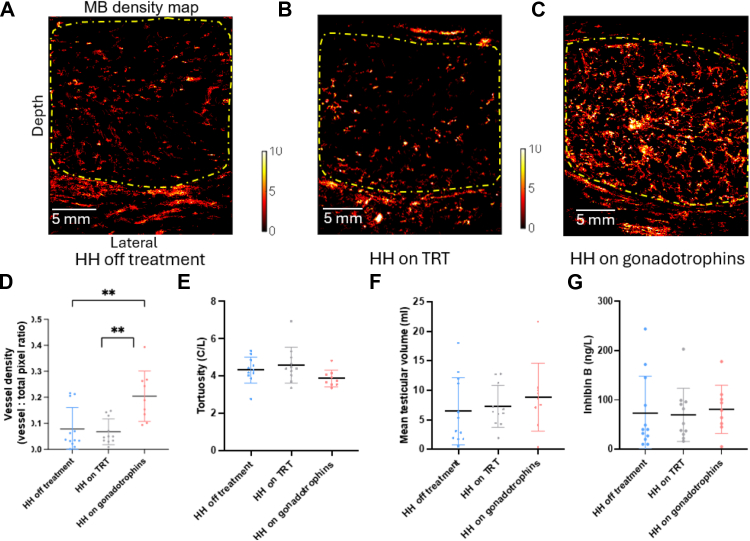
**ULM in hypogonadotrophic hypogonadism after gonadotrophin versus testosterone treatment.** (A–C): Super resolution images of super-localised MB in the longitudinal plane in men with HH who are treatment-naïve (A), treated with TRT (B), or treated with gonadotrophins (C). The intratesticular region of interest is marked with the yellow dotted line. (D–G) Column scatter plots demonstrating in the 3 study groups: treatment naïve, TRT treated, and gonadotrophin treated men with HH vessel density (D), tortuosity (E), testicular volume (F), inhibin B (G), plotted as mean ± standard deviation ∗∗p < 0.01, by one-way ANOVA, followed by Bonferroni’s post hoc analysis. N = 12 (men with HH off treatment), N = 11 (men with HH on TRT) and N = 9 (men with HH on gonadotrophins), except for (E) where N = 11 (men with HH off treatment). HH, hypogonadotrophic hypogonadism; TRT, testosterone replacement therapy; MB, microbubbles. The super resolution images from the transverse plane can be seen in Supplementary Fig. S17.

ULM showed greater vessel density with gonadotrophins (0.20 ± 0.10) versus untreated (0.08 ± 0.08, p = 0.003, by one way ANOVA) or TRT (0.07 ± 0.05, p = 0.002, by one way ANOVA) (Fig. 4A–D). Diameter was higher (98 ± 20 μm versus 77 ± 17 μm, p = 0.03 versus untreated, by one way ANOVA) and area greater (125 ± 104 mm^2^ versus 34 ± 30 mm^2^, p = 0.002 versus TRT, by one way ANOVA) (Supplementary Fig. S7). Tortuosity, testicular volume, inhibin B (Fig. 4E–G), and FRI (Supplementary Fig. S7) did not differ.

Vessel density and diameter correlated positively with testosterone (r = 0.69, 0.62; p < 0.001, by Spearman’s correlation) and inhibin B (r = 0.64, 0.65; p < 0.001, by Spearman’s correlation), whereas velocity correlated negatively with testosterone (r = −0.49, p < 0.01, by Spearman’s correlation) (Supplementary Figs. S8 and S9). Notably, vessel density and diameter were significantly higher in HH men with sperm compared to HH men with azoospermia (p < 0.001 and p < 0.01, respectively, by Mann–Whitney U test) (Supplementary Fig. S10).

### Study 3: longitudinal ULM in men with HH during treatment with gonadotrophins versus TRT

Men with HH treated with gonadotrophins or TRT (n = 7/group) underwent baseline (0-), 3-, 6-, and 12-month assessments. Flow diagram of the study participants can be found in Supplementary Fig. S11. Baseline characteristics were similar between the two groups, except for testicular volume (Table 3 and Supplementary Table S3). Two/group had sperm at baseline; after 12 months, 4/7 gonadotrophin-treated men had sperm. The completed semen analysis results are shown on Supplementary Table S4. Testosterone normalised by 6 months (Supplementary Fig. S12).

**Table 3:** Characteristics of men with hypogonadotrophic hypogonadism starting gonadotrophins versus testosterone replacement therapy.

Baseline characteristic (units)	Gonadotrophins n = 7	TRT n = 7	p value
Age (years)	37.6 ± 6.6 (31.4–43.7)	44 ± 11.8 (33.1–54.9)	0.24
BMI (kg/m^2^)	30.4 ± 7.9 (23.1–37.6)	29.1 ± 6.9 (22.7–35.6)	0.76
Testicular volume (ml)-median [percentiles]	2.8 [1.7, 5.3] (0.5–13.1)	6.4 [6, 7.2] (4.3–12.8)	0.04^a^
Hormonal profile			
Testosterone (nmol/L)	2.2 ± 1.7 (0.6–3.8)	3.4 ± 2.9 (0.7–6.1)	0.36
Oestradiol (pmol/L)			0.15
<100	7	5	
>100	0	2	
Inhibin B (ng/L)-median [percentiles]	32 [10, 40] (10–172)	52 [37, 96] (16–203)	0.20
Semen parameters			
Semen volume (ml)	1.5 [1, 2.3] (0.9–6.5)	3 [2.4, 4.5] (0.9–5.7)	0.13
Sperm concentration (millions/ml) -median [percentiles]	0.0 [0.0, 2.3] (0.0–43.0)	0.0 [0.0, 0.1] (0.0-0.1)	0.71
Azoospermia	5	5	>0.99
Sperm seen	2	2	

Continuous data are presented as mean ± standard deviation (95% CI) if normally distributed and median [IQR] (95% CI) if non normally distributed. Categorical data are presented as frequencies of counts.

BMI, body mass index; TRT, testosterone replacement therapy.

a Denotes where differences between groups were statistically significant at p < 0.05, by Student’s two-tailed t-test for parametric, Mann-Whitney U test for non-parametric data, Chi-Square test for categorical data.

**Fig. 5:**
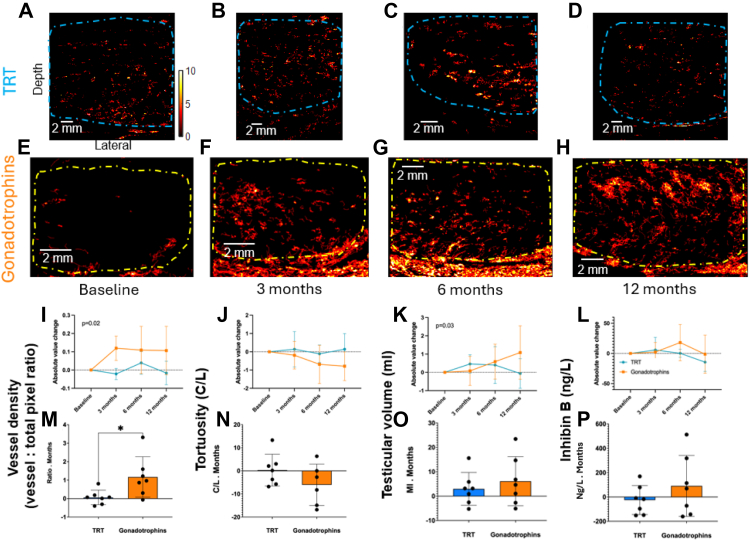
**Changes in the microvasculature in men with hypogonadotrophic hypogonadism before and during gonadotrophins therapy.** (A–D) Representative super resolution images obtained in the longitudinal plane from one participant on testosterone replacement therapy at baseline/0 month (A), 3 months (B), 6 months (C), and 12 months (D). The intratesticular region of interest is marked with the blue dotted line. (E–H) Representative super resolution images obtained in the longitudinal plane from one participant on gonadotrophins at baseline/0 month (E), 3 months (F), 6 months (G), and 12 months into treatment (H). The intratesticular region of interest is marked with the yellow dotted line. (I–P) Time profiles of change in (above), and mean area under the curve for select microvascular parameters and traditional markers of testicular function (below) in men with HH on TRT and men with HH on gonadotrophins. Vessel density (I), tortuosity (J), testicular volume (K), inhibin B (L) plotted as mean ± standard deviation on the y-axis, over the four time points on the x-axis. Inset is the results of the analysis of group by time interaction performed by two-way ANOVA/mixed effects model. ∗p < 0.05, by Student’s two-tailed t-test. N = 7 (men with HH on gonadotrophins) and N = 7 (men with HH on TRT), except for (J)& (N) where N = 6 (men with HH on gonadotrophins). MB, microbubbles; HH, hypogonadotrophic hypogonadism; TRT, testosterone replacement therapy. The super resolution images from the transverse plane can be seen in Supplementary Fig. S17.

Representative ULM images are shown in Fig. 5A–H. Mixed-effects modelling showed increases in vessel density (Fig. 5I), diameter, and area (Supplementary Fig. S13) with gonadotrophins versus TRT (p < 0.05, by mixed-effects model), but no differences in tortuosity (Fig. 5J), inhibin B (Fig. 5L), or FRI (Supplementary Fig. S13). Testicular volume rose more with gonadotrophins (p = 0.03, by two-way ANOVA) (Fig. 5K). Vessel density showed strongest discrimination between groups (AUC, p = 0.027, by ROC curve) (Fig. 5M−P).

### Study 4: pubertal blockade in rats

**Fig. 6:**
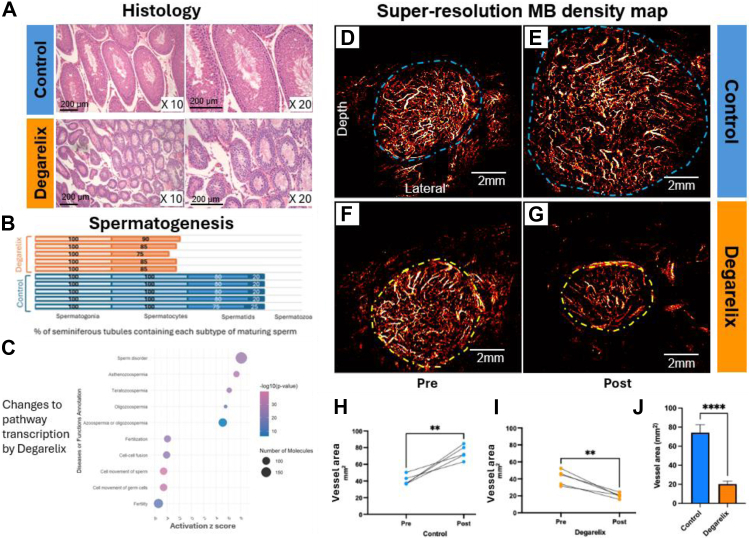
**Effect of pubertal blockade on microvascular development in rats.** (A): Longitudinal sections of the testis of a vehicle injected control and Degarelix-treated rodent, magnified ×10 and ×20, stained with haematoxylin and eosin. (B): Histological assessment of spermatogenesis of rodents treated with Degarelix and control. (C): Pathway analysis based on transcriptomic data from testicular tissue. (D–G): Super resolution images demonstrating MB density maps of the testis of a vehicle injected control (D, E) and a Degarelix-treated rodent (F, G), pre- (D, F) and post- (E, G) intervention; (H): Comparison of testicular vessel area of vehicle injected control pre- and post-intervention; (I): Comparison of testicular vessel area of a Degarelix-injected rodent pre- and post-intervention; (J): Comparison of testicular vessel area of Degarelix-injected rodent versus vehicle injected control. ∗∗∗∗, p < 0.0001; ∗∗, p < 0.01, by paired t test [(H), (I)] and Student’s two tailed t test [(J)]. N = 5 (Degarelix-injected rodents) and N = 5 (vehicle-control injected rodents). MB, microbubbles.

Juvenile male rats received Degarelix or vehicle. Baseline ULM parameters were similar (Supplementary Table S5). After 4 weeks, Degarelix blocked spermatogenesis which was confirmed histologically (Fig. 6A and B). Transcriptomics showed downregulation of germ cell/fertility pathways and upregulation of infertility pathways. Specifically, of 7184 genes, 6049 were downregulated and 1135 were upregulated. Fertilisation was the second most affected pathway (z-score −2.3). Within the disease and functions categorisation, the top altered annotations were all related to sperm dysfunction or fertilization abnormalities (Fig. 6C).

Rats received saline continue to grow as normally. The testicular AP diameter increased from 5.59 ± 0.58 mm to 9.43 ± 0.58 mm (p < 0.001, by student’s two tailed t-test) whereas it reduced in the Degarelix treated rats from 5.74 ± 0.39 mm to 3.95 ± 0.69 mm (p = 0.01, by student’s two tailed t-test) (Supplementary Fig. S14). Testis weight was 17-fold lower in Degarelix treated rats (0.093 ± 0.013 g versus 1.583 ± 0.049 g, p < 0.001, by student’s two tailed t-test). Supplementary Table S6 shows data on body weight, testes weight and size for both groups.

Representative ULM images from start and end of the study are shown in Fig. 6D–G. In normal puberty, vessel area increased but density fell with testis growth (Fig. 6H and Supplementary Fig. S15). Degarelix reduced vessel area (20.2 ± 3.1 mm^2^ versus 74.2 ± 8.5 mm^2^, p < 0.0001, by student’s two tailed t-test) and velocity (Fig. 6I and J and Supplementary Fig. S15). No other ULM parameters differed (Supplementary Fig. S15).

## Discussion

Reported global mean sperm counts have declined 55% since the 1970s, mirroring rising prevalences of comorbidities including obesity and type 2 diabetes.^5^ Together with the older mean age of female partners at attempted conception, this is driving rapid increases in the worldwide demand for fertility services.^50^ Routine investigations for testicular function do not always discriminate between male reproductive disorders and have only marginally progressed over the past couple of decades. Therefore, new tools are required to complement existing tools to further help patients with reproductive disorders.

In this work, we demonstrated the feasibility of ULM application in a readily available clinical scanner and mapping the testicular microvasculature with a resolution (90.3 um, estimated via Fourier ring correlation) higher than the classical US imaging resolution limit (128.3 um, defined by half-wavelength). The Fourier ring correlation curve from all ULM results can be found in the Supplementary Fig. S16. We have used ULM to identify discrepancies in the testicular microvasculature associated with testicular inactivation (HH) in men. HH is the deficient hypothalamo-pituitary stimulation of the testes, which causes low testosterone levels, male infertility, and pubertal failure if congenital. Our data reveal highly discrepant ULM microvascular parameters in men with HH compared with controls. Several ULM microvascular parameters such as vessel density, area, diameter and FRI were lower in men with HH compared with controls, reflecting testicular hypofunction. Vessel tortuosity was increased, which might be an adaptive response to tissue hypofunction or might reflect organ shrinkage in the absence of spermatogenesis. The preservation of mean microvascular flow velocity in individuals with HH, despite marked reductions in vessel density, diameter, total vessel area and flow volume, suggests that flow dynamics within the remaining perfused microvessels are largely maintained. Velocity reflects a local property of flow within individual vessels, whereas total perfusion is a network-level property dependent on the number and calibre of available conduits; thus, preserved velocity is compatible with reduced overall perfusion capacity due to microvascular rarefaction and/or remodelling rather than impaired flow within individual vessels. This interpretation is supported by prior ULM work, including the observation by Opacic et al.^25^ that mean microvascular velocity was remarkably similar across breast tumours with differing angiogenic phenotypes, and by consistent observations communicated by other ULM investigators that velocity varies little across experimental models. We acknowledge that velocity estimates derived from ULM are conditioned on the detection and tracking of sufficiently perfused MB, such that vessels with very fast, very low or intermittent flow may not contribute to velocity measurements, potentially obscuring subtle differences. Nevertheless, taken together, our findings are most consistent with a predominantly structural microvascular impairment, reflecting reduced microvascular capacity, while not excluding co-existing functional abnormalities such as impaired vasoreactivity, vessel recruitment or autoregulation, which would require dynamic or provocation-based assessments to detect.

HH is a unique model of male infertility owing to its potential for reversibility with prolonged gonadotrophin injections to emulate physiologic hypothalamo-pituitary function. We observed that the differences in ULM microvascular parameters in men with HH were ameliorated by gonadotrophin treatment compared with untreated or testosterone-treated men with HH. Finally, we have provided preliminary evidence that ULM detects restoration of testicular function in men with HH during gonadotrophin treatment long before currently available markers such as testicular volume or inhibin B are altered. 24 percent of men with HH remain azoospermic despite 2 years of gonadotrophin treatment for poorly understood reasons; treatment failure is devastating for affected couples by necessitating use of donor sperm, adoption, or acceptance of childlessness. Testicular volume improves (increases) during gonadotrophin treatment in HH and may predict successful spermatogenesis induction.^51^ Inhibin B, a gonadal peptide, is an established marker of Sertoli cell function and spermatogenesis in adults, with higher levels in fertile versus infertile men.^52^ Response of inhibin B levels to spermatogenesis induction tends to vary based on prior gonadotrophin exposure, state of seminiferous tubules development and pharmacological agents used.^53^^,^^54^ In our small longitudinal pilot study, testicular volume took 1 year to increase whilst inhibin B did not substantially change in men with HH during gonadotrophin treatment. However, ULM microvascular parameters appeared to increase just 3 months after commencing gonadotrophin treatment. Future studies could investigate whether ULM has the potential to personalise gonadotrophin treatment and provide earlier insight into the likelihood of successful sperm induction.

As for the clinical studies, a low frame rate clinical US scanner was used, which might be with limited capability for capturing fast moving MBs. We opted for a low tracking speed range (0–5 mm/s) to reduce erroneous MB tracks, considering that this velocity range corresponds to blood flow within capillaries and small arterioles.^55^ Previous studies have demonstrated the accuracy of the current microbubble tracking algorithm under low temporal resolution.^46^

We utilised a research scanner allowing for a higher number of frames per second compared to the clinical scanner and tracking faster moving MB (up to 25 mm/s) to investigate any microvascular alterations in a rodent model of pubertal blockade. It is worth noting that the human and rodent models are not broadly equivalent and therefore we would not necessarily expect the same outcomes. The differences observed in the microvascular parameters (decrease in vessel area in the Degarelix treated rats and increase in controls, respectively; decrease in vessel density in controls, but not in the Degarelix) was probably influenced by testis size. Controls have had normal pubertal development and as a result an increase in their testes size, whereas Degarelix kept the testes at pre pubertal state, but also resulted in further size reduction rather than no-progression of growth. Adult men with HH have no changes in their testicular volume. Across both species, there is a significant increase in the tubular compartment of the testis as a result of the expansion of the seminiferous tubules which are avascular, during puberty. The intertubular and peritubular small vessels run in the interstitial space between the tubules.^56^ The effects of Degarelix on adult rat testes had been described previously; 86% reduction in testicular weight was seen compared to vehicle injected controls at 45 days following a STAT 2 mg/kg Degarelix injection, which is similar to weight reductions observed in our study.^57^ The inclusion of histopathological analysis and transcriptomics allowed us to confirm the prepubertal state of Degarelix treated rats and further dissect into the effect of GnRH antagonist at a cellular level. Pathways associated with germ cell function and fertility were deactivated, and male infertility pathways were upregulated. Additionally, by using SVD in the image analysis process, some of the slow-moving MB had to be removed along with the tissue signal. Therefore, low velocity MB were not captured for the final quantification of the microvasculature. This might imply that low velocity blood flow assessment may be well-suited to assessing testicular function in men.

Functional MRI and nuclear scintigraphy allow spatial measurements of brain and endocrine organ functions by using a circulating tracer incorporated within cells in proportion to blood flow and metabolic activity. Accordingly, global testicular perfusion measured using Doppler, radioisotope or radioactive microspheres is known to increase during testicular activation in rodents.^58^^,^^59^ However, no prior study has directly investigated the relationship between testicular microvasculature and function. ULM has been used to detect structural cerebrovascular changes occurring during ageing,^60^ Alzheimer’s disease^61^ and stroke^62^ in rodent models, small vessel changes in tumorigenesis,^25^^,^^63^, ^64^, ^65^ and acute kidney injury^66^^,^^67^ among other applications. ULM has also been used to detect functional hyperaemia dynamically during brain activation.^68^ One previous study compared testicular ULM in men with obstructive and non-obstructive causes of oligo/azoospermia.^29^ However, its results are difficult to interpret since obstructive oligo/azoospermia is associated with testicular fluid accumulation which might affect ultrasound parameters. We have used ULM to provide functional relevance on the male gonad in a direct, detailed and spatially specific manner by using microvascular characteristics such as vessel area, density, flow and tortuosity. Schurich et al., by using CEUS demonstrated in a case report that testicular vessel density is decreased in a man with impaired testicular function compared to a man who had normal function.^69^ However, it is not specified the cause of impaired testicular function. Our study confirmed in a larger sample size and with a more robust technology that vessel density is lower in men with HH compared to healthy controls. Carlomagno et al., examined for differences in CEUS kinetic parameters between men with Klinefelter’s syndrome (primary hypogonadism) and healthy volunteers, and association with serum testosterone.^18^ Men with Klinefelter’s syndrome appeared to have irregular and chaotic vascular pattern on qualitative analysis which in line with our findings from men with HH. Testosterone appeared to correlate significantly, but mildly with CEUS kinetics (r: 0.31 to 0.43).^18^

B-mode ultrasound including ultrasound contrast agents are a routine, accessible and safe diagnostic tool for men investigated for testicular and reproductive disorders; software adaption of existing scanners would allow ULM-measurement of testicular function. Deficiency of serum testosterone, the gold-standard measurement for Leydig-cell testicular function, is closely correlated with clinical hypogonadism. However, serum testosterone is a poor marker for recovery of testicular function in conditions such as hypogonadotrophic hypogonadism or androgen abuse. Semen analysis provides information about fertility potential, but retrograde ejaculation, acute illness, and personal or cultural inhibition make it unsuitable in some situations. ULM might provide a screening tool to guide fertility management where semen analysis cannot be performed. ULM could also guide prognosis for successful surgical sperm retrieval in men acutely ill with cancer. Currently, ULM requires the analysis of MB during CEUS. However, it is possible that ULM could be adapted to analyse erythrocytes without the need for contrast injection.^70^

Our study is strengthened by its use of canonical models of testicular inactivation (pubertal blockade and HH) to demonstrate the ability of ULM to assess testicular function in rodents and humans. Our cohort of men with HH had organic hypothalamic or pituitary level defects either of congenital or acquired aetiology. Consistent with clinical guidelines, all participants had unequivocally low pre-treatment serum levels of LH, FSH and testosterone. The ULM processing was performed by a team who was blinded to the clinical information of each study subject. The US was performed by a single clinician/scientist to minimise interobserver variability and the ROI was drawn by a different clinician in a blinded manner. The studies suffer from certain limitations. Firstly, CEUS techniques, such as microbubble intensity measurements and TIC analysis were not utilised in this study and therefore remain unknown if the observed differences in testicular vascularity could have also been captured by conventional CEUS. However, ULM is considered a much more robust technique to map the microvasculature, with challenges on quantifying CEUS being described previously.^19^ ULM benefits from MB tracking and more quantitative information of the rich microvascular details could be generated and quantified.^71^ Secondly, direct comparison between the clinical and rodent studies cannot be made owing to the use of different ultrasound systems with different frame rates, imaging sequence, probes, contrast agents and different disease models. Our aim was to explore the utility of ULM to help understand pathophysiological differences in the testes in various reproductive disorders.

Additionally, it should be noted that each ULM image shows microvasculature representing a limited sample of the vast vascular network in the organ. As each such image was generated by accumulating microbubble signals over multiple sequential image frames, the degree of saturation in each sampled vessels depends on the number of total image frames and the length of the acquisition time, but the data acquisition process was standardised and kept constant throughout the clinical studies. Future studies can additionally incorporate within subject normalisation to provide a more accurate assessment of the vascular density. We used average data from both planes and testes, and therefore we were not in a position to study lateralisation of function, nevertheless there was no clinical suspicion that we would have found that. Future studies could investigate whether there are differences in these parameters between testes, although this has not been described in the absence of testicular volume discrepancy. In human studies, clinical acquisition was limited by the scanner used which has relatively low frame rate (13 fps) and limited capacity to store images in a single imaging session. This results in reduced vascular saturation in our ULM images and likely contributes to the variabilities observed in the final quantification results. Given that higher frame rate imaging systems are emerging in the clinic, future translation of the technique using a high frame rate system and longer acquisition should generate more saturated images and demonstrate even more robust correlations between the ULM based image markers and clinical markers in men. Another limitation of this study is the inherent challenge of acquiring the exact same imaging plane in every subject. Nevertheless, the testicular vascular architecture is relatively homogeneous, which supports the comparability of vascular parameters sampled from standardised imaging planes across individuals. Importantly, despite potential variability arising from sampling slightly different planes, we were still able to detect consistent differences between patient groups, suggesting that the observed findings are robust to this source of variation. Our studies are pilot and exploratory with a small sample size that precludes multivariate analysis to adjust for covariates. Lastly, our study lacks mechanistic explanation.

These proof-of-concept findings require larger further studies to better define the diagnostic potential of ULM compared with existing diagnostic markers in clinical practice. We included men with HH who had either normal or deficient pubertal development; future comparative studies may investigate whether ULM microvascular profiles differ between these subgroups. Furthermore, the results of our study cannot be extrapolated to other male reproductive disorders such as primary hypogonadism or idiopathic infertility. Our small, pilot cohort study suggests that ULM markers improve more rapidly compared to traditional testicular markers (testicular volume) during gonadotrophin treatment in men with HH. An adequately powered study would be needed to investigate whether normalisation of ULM markers correlates with successful induction of spermatogenesis during gonadotrophin treatment.

In summary, we suggest that ULM is a diagnostic modality allowing the study of previously undetectable microvascular characteristics correlating with tissue function. Therefore, ULM has potential as a novel avenue for studying organ function and disease. Promisingly, ULM microvascular markers normalise in a more pronounced and rapid manner compared with clinical, biochemical or seminal markers in men commensurate with physiological restoration of testicular function. Larger studies are needed to explore the clinical potential of ULM to enhance the management of male infertility and other disorders of organ function.

## Contributors

Conceptualisation: CNJ, MXT.

Methodology: CNJ, MXT, AL, KM, NP.

Rodent studies: CD, PG, JBV.

Transcriptome analysis: GB.

Investigation and study visits: NP, CG, RD, NDS.

Contrast enhanced ultrasound: AL.

ULM analysis: JY, BH.

Access and verify the underlying data: JY, BH, NP, MXT, CNJ.

Data and statistical analysis: NP, RD.

Writing—original draft: RD, NDS, NP.

Writing—review and editing: JY, BH, KM, AL, SM, RL, WD, MXT, CNJ.

Supervision: CNJ, MXT.

All authors read and approved the final version of the manuscript.

## Data sharing statement

Data that support the findings of this project are available from the corresponding authors upon reasonable request.

## Declaration of interests

Mengxing Tang is a member of the Scientific Advisory Board of Verasonics Ltd and a minority shareholder in Sonalis Imaging Ltd and Cardioacc Ltd. These affiliations are not related to this work. The remaining authors declare no conflicts of interest.

## Appendix A Supplementary data

Supplementary Figs. S1–S17, Tables S1–S6, and Videos S1 and S2

## References

[1] De Silva N.L., Papanikolaou N., Grossmann M. (2024). Male hypogonadism: pathogenesis, diagnosis, and management. Lancet Diabetes Endocrinol.

[2] Zarotsky V., Huang M.Y., Carman W. (2014). Systematic literature review of the risk factors, comorbidities, and consequences of hypogonadism in men. Andrology.

[3] Mann U., Shiff B., Patel P. (2020). Reasons for worldwide decline in male fertility. Curr Opin Urol.

[4] Levine H., Jørgensen N., Martino-Andrade A. (2017). Temporal trends in sperm count: a systematic review and meta-regression analysis. Hum Reprod Update.

[5] Levine H., Jørgensen N., Martino-Andrade A. (2023). Temporal trends in sperm count: a systematic review and meta-regression analysis of samples collected globally in the 20th and 21st centuries. Hum Reprod Update.

[6] Skakkebaek N.E., Rajpert-De Meyts E., Buck Louis G.M. (2016). Male reproductive disorders and fertility trends: influences of environment and genetic susceptibility. Physiol Rev.

[7] Choy J.T., Eisenberg M.L. (2018). Male infertility as a window to health. Fertil Steril.

[8] Gerris J. (1999). Methods of semen collection not based on masturbation or surgical sperm retrieval. Hum Reprod Update.

[9] Bahk J.Y., Jung J.H., Jin L.M., Min S.K. (2010). Cut-off value of testes volume in young adults and correlation among testes volume, body mass index, hormonal level, and seminal profiles. Urology.

[10] Condorelli R., Calogero A.E., La Vignera S. (2013). Relationship between testicular volume and conventional or nonconventional sperm parameters. Int J Endocrinol.

[11] Ehala-Aleksejev K., Punab M. (2018). Relationships between total testicular volume, reproductive parameters and surrogate measures of adiposity in men presenting for couple’s infertility. Andrologia.

[12] Garolla A., Grande G., Palego P. (2021). Central role of ultrasound in the evaluation of testicular function and genital tract obstruction in infertile males. Andrology.

[13] Xu C., Liu M., Zhang F.F. (2014). The association between testicular microlithiasis and semen parameters in Chinese adult men with fertility intention: experience of 226 cases. Urology.

[14] Pozza C., Kanakis G., Carlomagno F. (2020). Testicular ultrasound score: a new proposal for a scoring system to predict testicular function. Andrology.

[15] Hillelsohn J.H., Chuang K.W., Goldenberg E., Gilbert B.R. (2013). Spectral doppler sonography: a noninvasive method for predicting dyspermia. J Ultrasound Med.

[16] Schlegel P.N., Sigman M., Collura B. (2021). Diagnosis and treatment of infertility in men: AUA/ASRM guideline part I. Fertil Steril.

[17] Mittal P.K., Little B., Harri P.A. (2017). Role of imaging in the evaluation of Male infertility. Radiographics.

[18] Carlomagno F., Pozza C., Tenuta M. (2022). Testicular microvascular flow is altered in Klinefelter Syndrome and predicts circulating testosterone. J Clin Endocrinol Metab.

[19] Tang M.X., Mulvana H., Gauthier T. (2011). Quantitative contrast-enhanced ultrasound imaging: a review of sources of variability. Interface Focus.

[20] Dencks S., Schmitz G. (2023). Ultrasound localization microscopy. Z Med Phys.

[21] Christensen-Jeffries K., Couture O., Dayton P.A. (2020). Super-resolution ultrasound imaging. Ultrasound Med Biol.

[22] Errico C., Pierre J., Pezet S. (2015). Ultrafast ultrasound localization microscopy for deep super-resolution vascular imaging. Nature.

[23] Christensen-Jeffries K., Browning R.J., Tang M.X., Dunsby C., Eckersley R.J. (2015). In vivo acoustic super-resolution and super-resolved velocity mapping using microbubbles. IEEE Trans Med Imaging.

[24] Song P., Rubin J.M., Lowerison M.R. (2023). Super-resolution ultrasound microvascular imaging: is it ready for clinical use?. Z Med Phys.

[25] Opacic T., Dencks S., Theek B. (2018). Motion model ultrasound localization microscopy for preclinical and clinical multiparametric tumor characterization. Nat Commun.

[26] Zhu J., Zhang C., Christensen-Jeffries K. (2022). Super-resolution ultrasound localization microscopy of microvascular structure and flow for distinguishing metastatic lymph nodes - an initial human study. Ultraschall der Medizin.

[27] Demené C., Robin J., Dizeux A. (2021). Transcranial ultrafast ultrasound localization microscopy of brain vasculature in patients. Nat Biomed Eng.

[28] Yan J., Huang B., Tonko J. (2024). Transthoracic ultrasound localization microscopy of myocardial vasculature in patients. Nat Biomed Eng.

[29] Li M., Zhang X., Yan J. (2024). Non-invasive ultrasound localization microscopy (ULM) in azoospermia: connecting testicular microcirculation to spermatogenic functions. Theranostics.

[30] Bangash M.N., Pearse R.M., Vincent J.L., Hall J.B. (2012). Microcirculation BT - encyclopedia of intensive care medicine.

[31] Pries A.R., Reglin B., Secomb T.W. (2001). Structural adaptation of microvascular networks: functional roles of adaptive responses. Am J Physiol Heart Circ Physiol.

[32] Damber J.E., Bergh A. (1992). Testicular microcirculation—a forgotten essential in andrology?. Int J Androl.

[33] Herwig R., Tosun K., Pinggera G.M. (2004). Tissue perfusion essential for spermatogenesis and outcome of testicular sperm extraction (TESE) for assisted reproduction. J Assist Reprod Genet.

[34] Damber J.E., Janson P.O. (1978). Testicular blood flow and testosterone concentration in spermatic venous blood of anaesthetized rats. J Reprod Fertil.

[35] Collin O., Bergh A., Damber J.E., Widmark A. (1993). Control of testicular vasomotion by testosterone and tubular factors in rats. J Reprod Fertil.

[36] Rebourcet D., Wu J., Cruickshanks L. (2016). Sertoli cells modulate testicular vascular network development, structure, and function to influence circulating testosterone concentrations in adult Male mice. Endocrinology.

[37] Papanikolaou N., Luo R., Jayasena C.N. (2022). Fertility considerations in hypogonadal men. Endocrinol Metab Clin North Am.

[38] Jayasena C.N., Anderson R.A., Llahana S. (2022). Society for endocrinology guidelines for testosterone replacement therapy in male hypogonadism. Clin Endocrinol (Oxf).

[39] World Health Organization (2021).

[40] Kim N.R., Khalil R., David K. (2021). Novel model to study the physiological effects of temporary or prolonged sex steroid deficiency in male mice. Am J Physiol Endocrinol Metab.

[41] Hansen-Shearer J., Yan J., Lerendegui M. (2024). Ultrafast 3-D transcutaneous super resolution ultrasound using row-column array specific coherence-based beamforming and rolling acoustic sub-aperture processing: in vitro, in rabbit and in human study. Ultrasound Med Biol.

[42] Sullender C.T., Richards L.M., He F., Luan L., Dunn A.K. (2022). Dynamics of isoflurane-induced vasodilation and blood flow of cerebral vasculature revealed by multi-exposure speckle imaging. J Neurosci Methods.

[43] Baranger J., Arnal B., Perren F., Baud O., Tanter M., Demene C. (2018). Adaptive spatiotemporal SVD clutter filtering for ultrafast doppler imaging using similarity of spatial singular vectors. IEEE Trans Med Imaging.

[44] Brown J., Christensen-Jeffries K., Harput S. (2019). Investigation of microbubble detection methods for super-resolution imaging of microvasculature. IEEE Trans Ultrason Ferroelectr Freq Control.

[45] Harput S., Christensen-Jeffries K., Brown J. (2018). Two-stage motion correction for super-resolution ultrasound imaging in human lower limb. IEEE Trans Ultrason Ferroelectr Freq Control.

[46] Yan J., Zhang T., Broughton-Venner J., Huang P., Tang M.X. (2022). Super-resolution ultrasound through sparsity-based deconvolution and multi-feature tracking. IEEE Trans Med Imaging.

[47] Hingot V., Chavignon A., Heiles B., Couture O. (2021). Measuring image resolution in ultrasound localization microscopy. IEEE Trans Med Imaging.

[48] Taghavi I., Andersen S.B., Hoyos C.A.V., Nielsen M.B., Sorensen C.M., Jensen J.A. (2021). In vivo motion correction in super-resolution imaging of rat kidneys. IEEE Trans Ultrason Ferroelectr Freq Control.

[49] Julious S.A. (2005). Sample size of 12 per group rule of thumb for a pilot study. Pharm Stat.

[50] Ubaldi F.M., Cimadomo D., Vaiarelli A. (2019). Advanced maternal age in IVF: still a challenge? The present and the future of its treatment. Front Endocrinol (Lausanne).

[51] Young J., Xu C., Papadakis G.E. (2019). Clinical management of congenital hypogonadotropic hypogonadism. Endocr Rev.

[52] Kumanov P., Nandipati K., Tomova A., Agarwal A. (2006). Inhibin B is a better marker of spermatogenesis than other hormones in the evaluation of male factor infertility. Fertil Steril.

[53] Meachem S.J., Nieschlag E., Simoni M. (2001). Inhibin B in male reproduction: pathophysiology and clinical relevance. Eur J Endocrinol.

[54] Kolb B.A., Stanczyk F.Z., Sokol R.Z. (2000). Serum inhibin B levels in males with gonadal dysfunction. Fertil Steril.

[55] Piechnik S.K., Chiarelli P.A., Jezzard P. (2008). Modelling vascular reactivity to investigate the basis of the relationship between cerebral blood volume and flow under CO2 manipulation. Neuroimage.

[56] Weerasooriya T.R., Yamamoto T. (1985). Three-dimensional organisation of the vasculature of the rat spermatic cord and testis. A scanning electron-microscopic study of vascular corrosion casts. Cell Tissue Res.

[57] Broqua P., Riviere P.J.M., Michael Conn P., Rivier J.E., Aubert M.L., Junien J.L. (2002). Pharmacological profile of a new, potent, and long-acting gonadotropin-releasing hormone antagonist: degarelix. J Pharmacol Exp Ther.

[58] Setchell B.P., Sharpe R.M. (1981). Effect of injected human chorionic gonadotrophin on capillary permeability, extracellular fluid volume and the flow of lymph and blood in the testes of rats. J Endocrinol.

[59] Geesaman B., Villanueva-Meyer J., Bluestein D., Miller L., Mena I., Rajfer J. (1992). Effects of multiple injections of HCG on testis blood flow. Urology.

[60] Lowerison M.R., Sekaran N.V.C., Zhang W. (2022). Aging-related cerebral microvascular changes visualized using ultrasound localization microscopy in the living mouse. Sci Rep.

[61] Lowerison MR, Vaithiyalingam Chandra Sekaran N, Dong Z (2024). Super-resolution ultrasound reveals cerebrovascular impairment in a mouse model of Alzheimer’s disease. J Neurosci.

[62] Chavignon A., Hingot V., Orset C., Vivien D., Couture O. (2022). 3D transcranial ultrasound localization microscopy for discrimination between ischemic and hemorrhagic stroke in early phase. Sci Rep.

[63] Lowerison M.R., Huang C., Lucien F., Chen S., Song P. (2020). Ultrasound localization microscopy of renal tumor xenografts in chicken embryo is correlated to hypoxia. Sci Rep.

[64] Lin F., Shelton S.E., Espíndola D., Rojas J.D., Pinton G., Dayton P.A. (2017). 3-D ultrasound localization microscopy for identifying microvascular morphology features of tumor angiogenesis at a resolution beyond the diffraction limit of conventional ultrasound. Theranostics.

[65] Zhang W., Lowerison M.R., Dong Z., Miller R.J., Keller K.A., Song P. (2021). Super-resolution ultrasound localization microscopy on a rabbit liver VX2 tumor model: an initial feasibility study. Ultrasound Med Biol.

[66] Chen Q., Yu J., Rush B.M., Stocker S.D., Tan R.J., Kim K. (2020). Ultrasound super-resolution imaging provides a noninvasive assessment of renal microvasculature changes during mouse acute kidney injury. Kidney Int.

[67] Andersen S.B., Taghavi I., Hoyos C.A.V. (2020). Super-resolution imaging with ultrasound for visualization of the renal microvasculature in rats before and after renal ischemia: a pilot study. Diagnostics.

[68] Renaudin N., Demené C., Dizeux A., Ialy-Radio N., Pezet S., Tanter M. (2022). Functional ultrasound localization microscopy reveals brain-wide neurovascular activity on a microscopic scale. Nat Methods.

[69] Schurich M., Aigner F., Frauscher F., Pallwein L. (2009). The role of ultrasound in assessment of male fertility. Eur J Obstet Gynecol Reprod Biol.

[70] Arendt Jensen J., Amin Naji M., Kazmarek Praesius S. (2024). Super-resolution ultrasound imaging using the erythrocytes-part I: density images. IEEE Trans Ultrason Ferroelectr Freq Control.

[71] Dencks S., Lowerison M., Hansen-Shearer J. (2025). Super-resolution ultrasound: from data acquisition and motion correction to localization, tracking, and evaluation. IEEE Trans Ultrason Ferroelectr Freq Control.

