# Supplementary material for: Ultrasound localisation microscopy tracks testicular microvascular adaptations to endocrine function in male infertility
Source: eBioMedicine. 2026 Jun 18;129:106333. doi: 10.1016/j.ebiom.2026.106333 (PMC13310648; doi:10.1016/j.ebiom.2026.106333)
Supplement: Supplementary Figs. S1–S17, Tables S1–S6, and Videos S1 and S2 [file mmc1.docx]

**Supplementary figures**

**Figure S1.
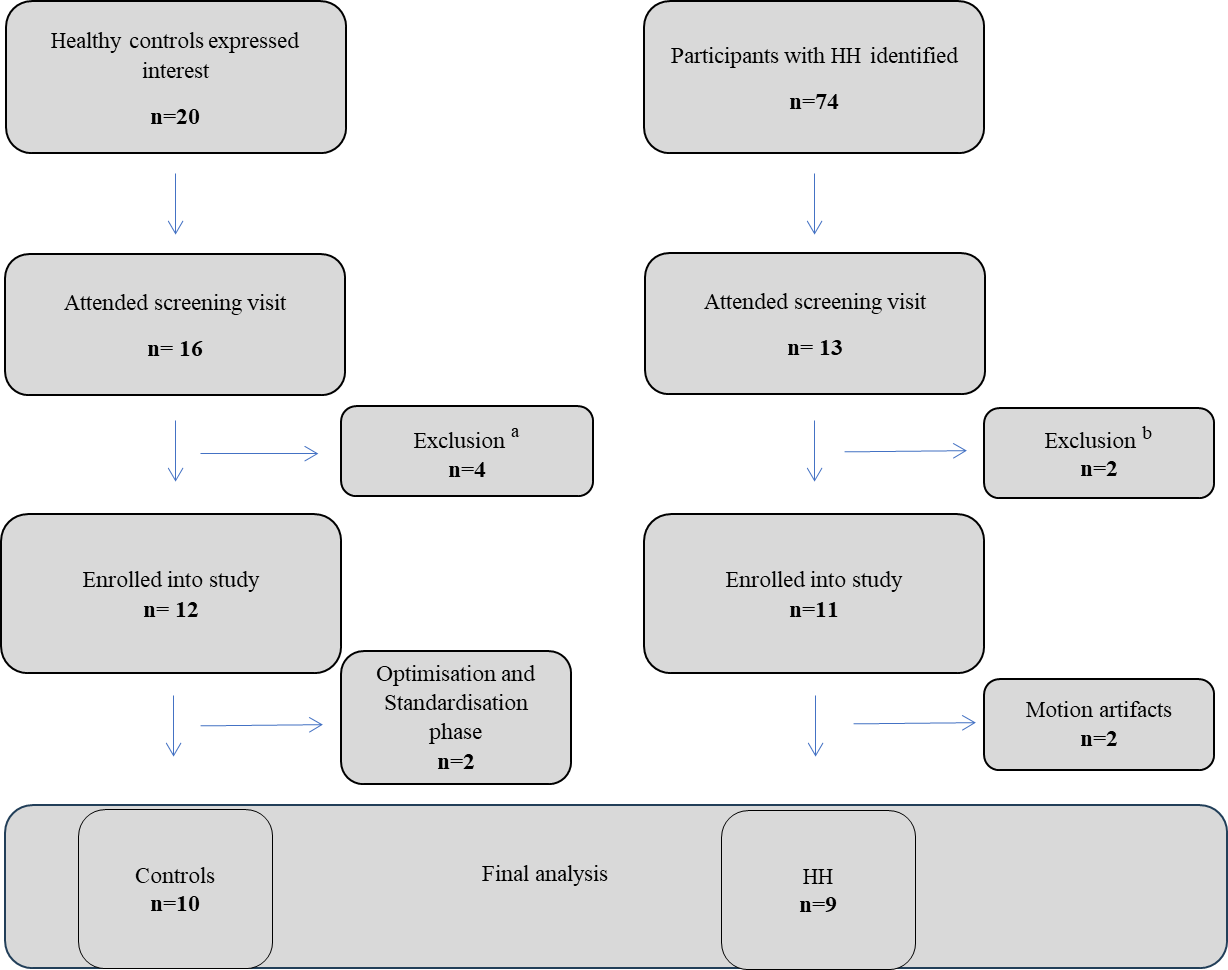
Study 1 flow chart**

HH: Hypogonadotrophic hypogonadism.

^a^ reasons for exclusion: borderline serum testosterone, n=1; azoospermia, n=1; loss to follow up, n=1; acute illness, n=1.

^b^ reasons for exclusion: serum testosterone >8 nmol/L, n=2

**
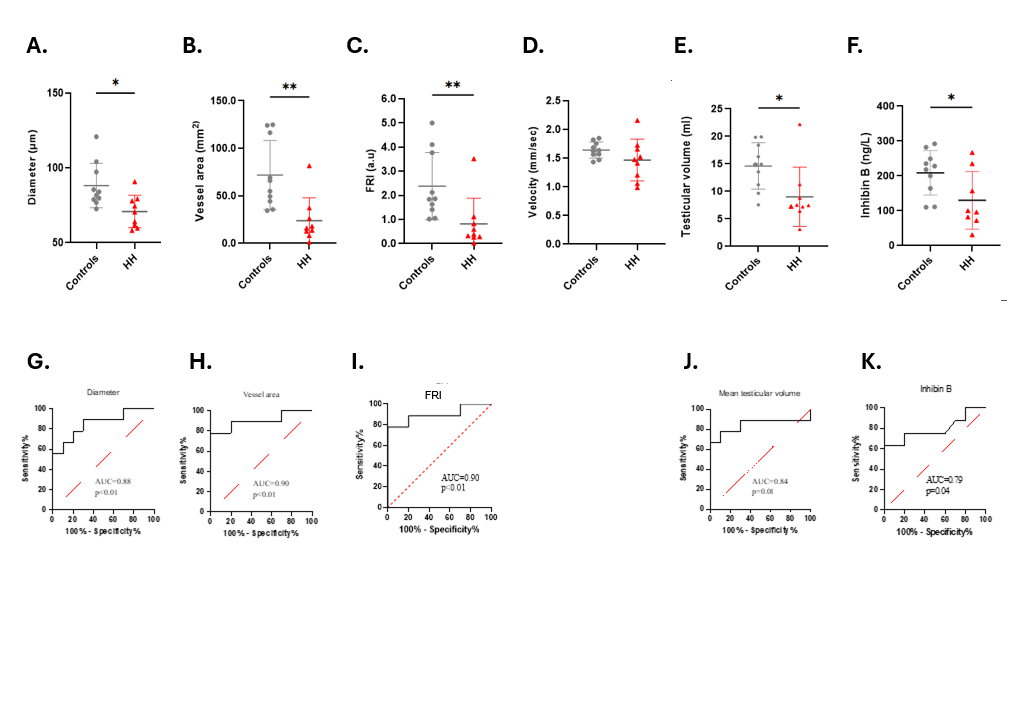
**

**Figure S2. Comparing microvascular characteristics between men with hypogonadotrophic hypogonadism versus controls**

**(A-F)** Column scatter plots of select microvascular parameters as calculated with super resolution image processing, and traditional markers of testicular function in controls and men with hypogonadotrophic hypogonadism, diameter **(A)**, vessel area **(B)**, FRI **(C)**, velocity **(D)**, testicular volume **(E)**, inhibin B **(F)**. Data is displayed as mean ± standard deviation. * p<0·05, ** p<0·01, by Student’s two-tailed t-test or Mann-Whitney U test for parametric and non-parametric data, respectively. N=9 (men with HH) and N=10 (healthy men), except for (F) where N=8 (men with HH)

**(G-K)** Receiver operating characteristic curves for select microvascular parameters as calculated with super resolution image processing, and traditional markers of testicular function in controls and men with hypogonadotrophic hypogonadism, diameter **(G)**, vessel area **(H)**, FRI **(I)**, testicular volume **(J)**, inhibin B **(K).** AUC and level of significance are shown on the graphs.

FRI, flow related index; a.u, arbitrary units; HH, hypogonadotrophic hypogonadism

**
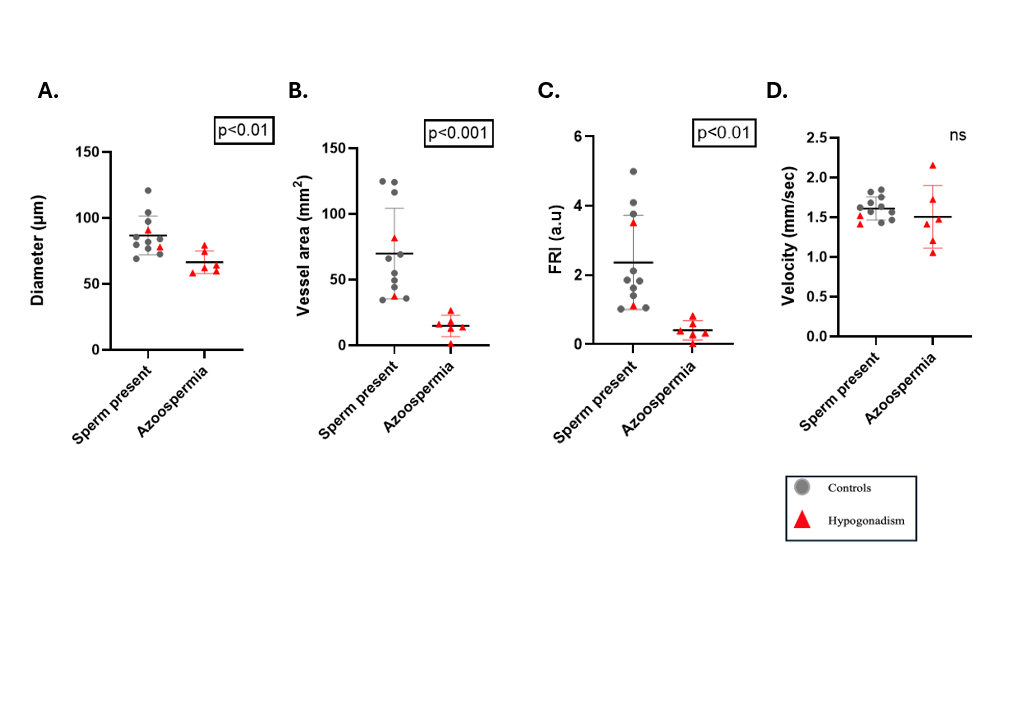
**

**Figure S3. Comparing microvascular characteristics between men with sperm and without sperm**

**(A-D)** Column scatter plots of select microvascular parameters as calculated with super resolution image processing, diameter **(A)**, vessel area **(B)**, FRI **(C)**, velocity **(D)** in men with and without azoospermia. Data is displayed as mean ± standard deviation. p values are calculated by Student’s two-tailed t-test or Mann-Whitney U test for parametric and non-parametric data, respectively. N=8 (men with HH) and N=10 (healthy men)

FRI, flow related index; a.u, arbitrary units

 
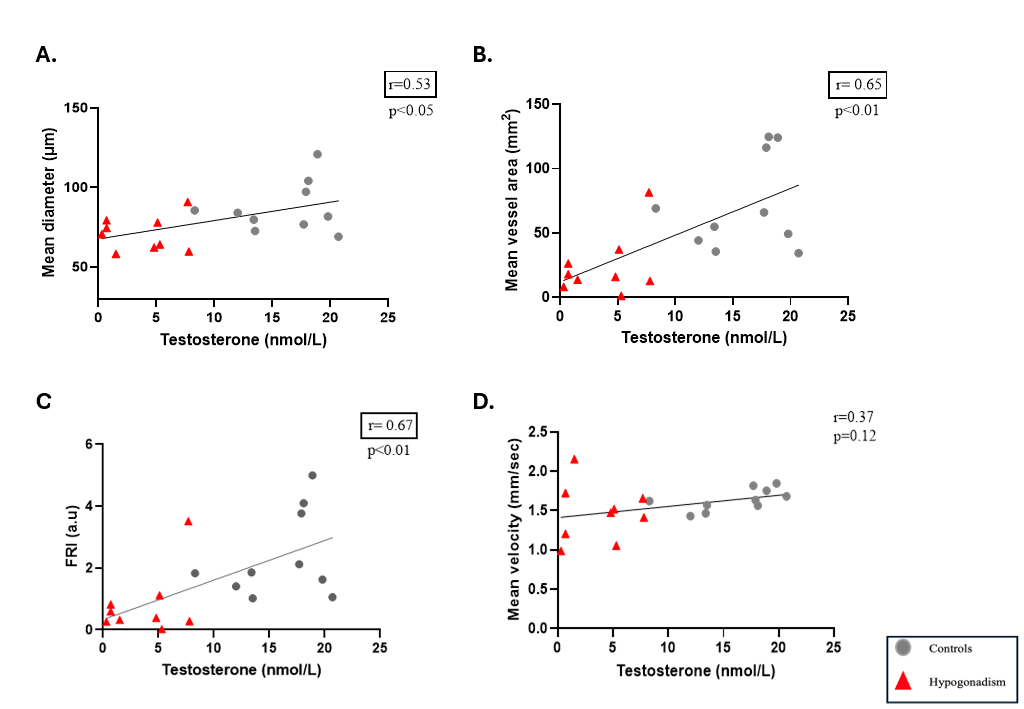


**Figure S4. Relationships between serum testosterone and microvascular characteristics in men with hypogonadotrophic hypogonadism and controls**

**(A-D)** Correlation between serum testosterone and diameter **(A)**, vessel area **(B)**, FRI **(C)**, velocity **(D)**. Correlations were calculated with Pearson correlation coefficient or Spearman’s rank correlation coefficient for parametric and non-parametric data, respectively. Degree of association and p value are shown on the plots. N=9 (men with HH) and N=10 (healthy men)

FRI, flow related index; a.u, arbitrary units

  
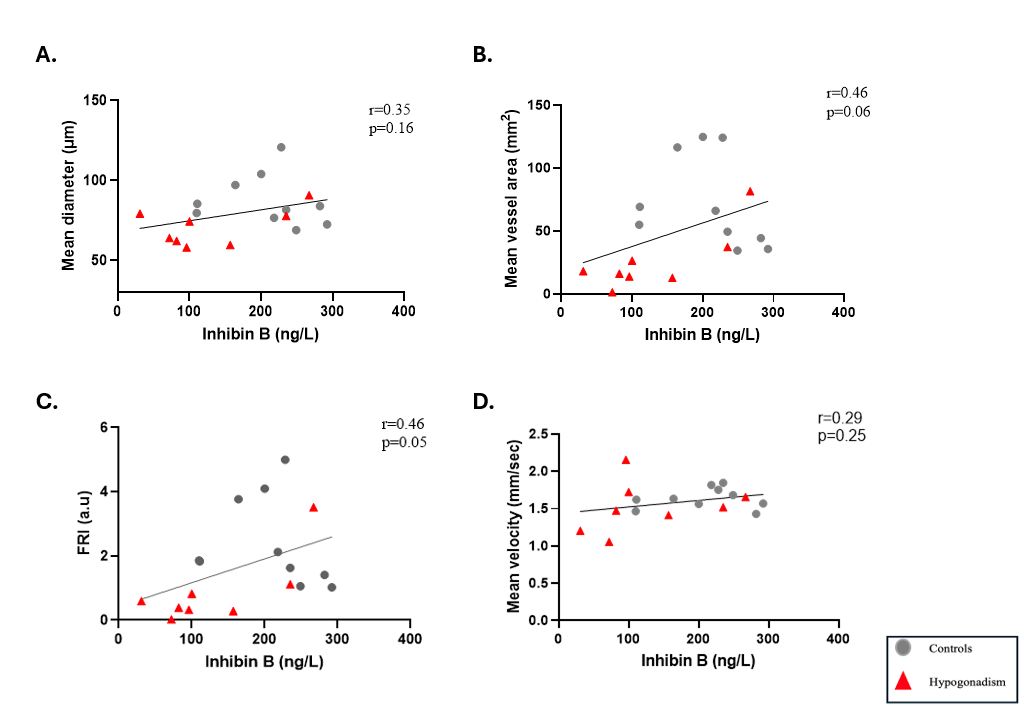


**Figure S5. Relationships between serum inhibin B and microvascular characteristics in men with hypogonadotrophic hypogonadism and controls**

**(A-D)** Correlation between serum inhibin B and diameter **(A)**, vessel area **(B)**, FRI **(C)**, velocity **(D)**. Correlations were calculated with Pearson correlation coefficient or Spearman’s rank correlation coefficient for parametric and non-parametric data, respectively. Degree of association and p value are shown on the plots. N=8 (men with HH) and N=10 (healthy men)

FRI, flow related index; a.u, arbitrary units


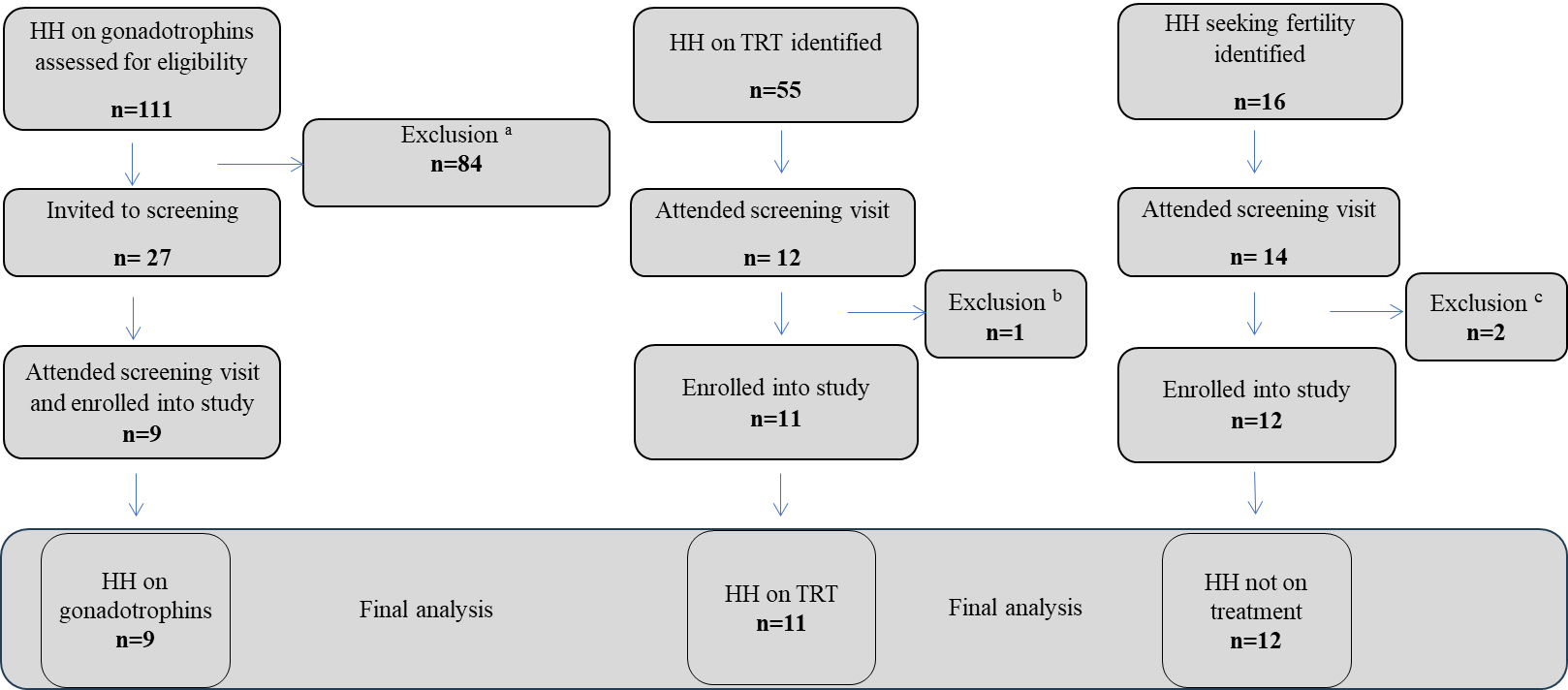


**Figure S6. Study 2 flow chart**

HH: hypogonadotrophic hypogonadism; TRT: testosterone replacement therapy.

^a^ reason for exclusion: no longer on gonadotrophins, n=35; previous testicular surgery, n= 6; primary testicular failure, n= 19; unable to contact, n=6; age<18 years, n= 3; age >60 years, n= 2; other, n= 13

^b^ reason for exclusion: not interested, n=1

^c^ reason for exclusion: not diagnosed with HH, n=2


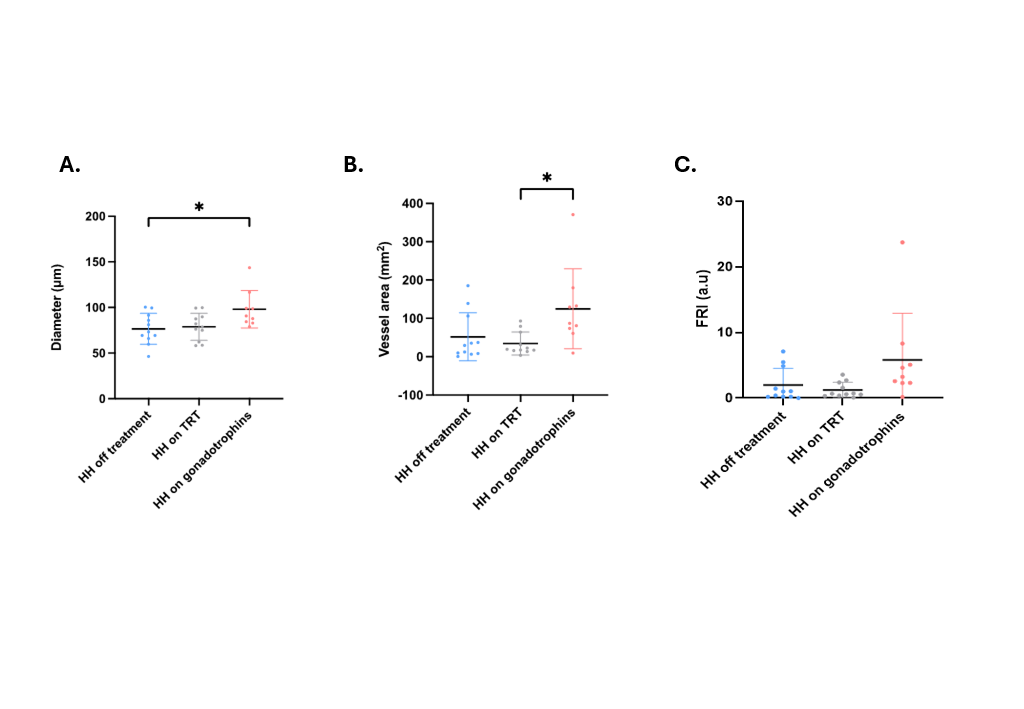


**Figure S7. Comparing microvascular characteristics in men with hypogonadotrophic hypogonadism following gonadotrophin versus testosterone treatment.**

**(A-D)** Column scatter plots comparing parameters in men with HH who are treatment naïve, on TRT or gonadotrophins: Vessel diameter (**A**), area (**B**), FRI (**C**) plotted as mean ± standard deviation

* p<0·05, ** p<0·01, by one-way ANOVA, followed by Bonferroni’s post hoc analysis. N=11 (men with HH off treatment), N=11 (men with HH on TRT) and N=9 (men with HH on gonadotrophins)

FRI, flow related index; a.u, arbitrary units; HH, hypogonadotrophic hypogonadism; TRT, testosterone replacement therapy

 
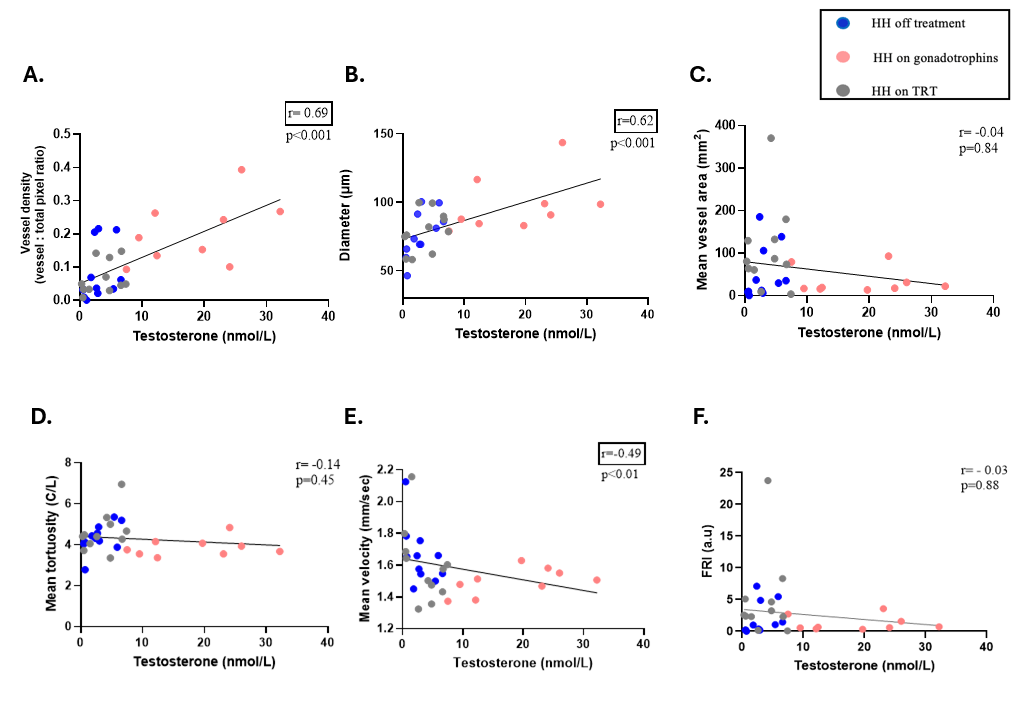


**Figure S8. Relationships between serum testosterone and microvascular characteristics in men with hypogonadotrophic hypogonadism on gonadotrophins and testosterone treatment.**

**(A-F)** Scatter plot correlation between serum testosterone and vessel density **(A)**, diameter **(B)**, vessel area **(C)**, tortuosity **(D)**, velocity **(E)**, FRI **(F)**.

Correlations were calculated with Pearson correlation coefficient or Spearman’s rank correlation coefficient for parametric and non-parametric data, respectively. Degree of association and p value are shown on the plots. N=11 (men with HH off treatment), N=11 (men with HH on TRT) and N=9 (men with HH on gonadotrophins), except for (A) where N=12 (men with HH off treatment)

FRI, flow related index; a.u, arbitrary units; HH, hypogonadotrophic hypogonadism; TRT, testosterone replacement therapy

 
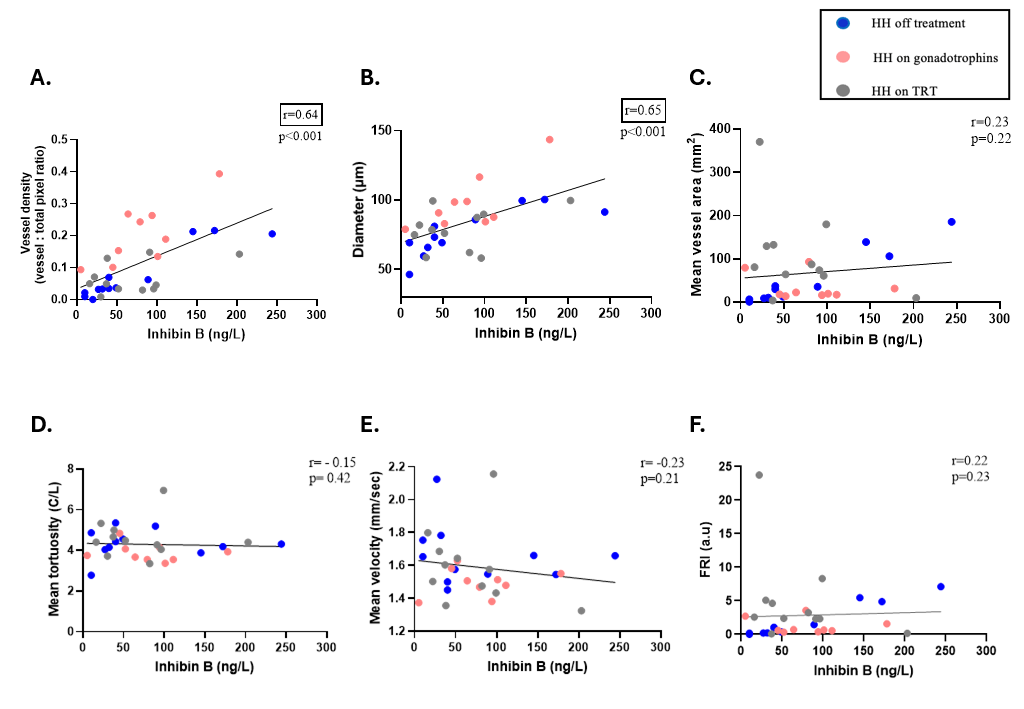


**Figure S9. Relationships between serum inhibin B and microvascular** **characteristics in men with hypogonadotrophic hypogonadism on gonadotrophins and testosterone treatment.**

**(A-F)** Scatter plot correlation between serum inhibin B and vessel density **(A)**, diameter **(B)**, vessel area **(C)**, tortuosity **(D)**, velocity **(E)**, FRI **(F)**. Correlations were calculated with Pearson correlation coefficient or Spearman’s rank correlation coefficient for parametric and non-parametric data, respectively. Degree of association and p value are shown on the plots. N=11 (men with HH off treatment), N=11 (men with HH on TRT) and N=9 (men with HH on gonadotrophins), except for (A) where N=12 (men with HH off treatment).

FRI, flow related index; a.u, arbitrary units; HH, hypogonadotrophic hypogonadism; TRT, testosterone replacement therapy

  
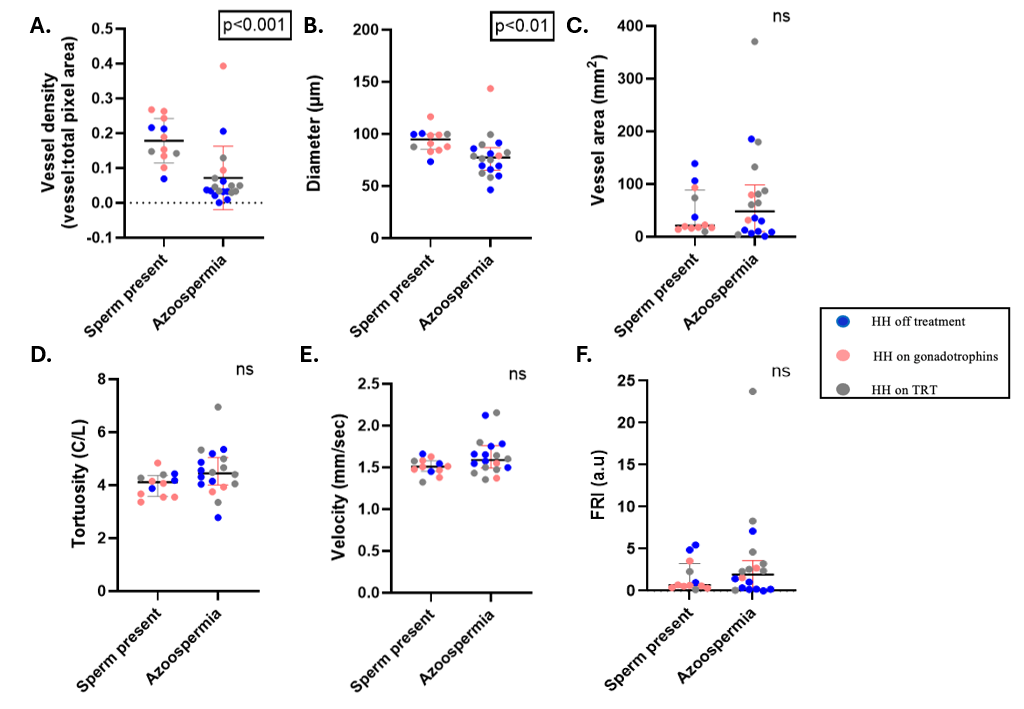


**Figure S10. Comparing microvascular characteristics in HH men with sperm and without sperm on no treatment, gonadotrophins and testosterone.**

**(A-F)** Column scatter plots of select microvascular parameters as calculated with super resolution image processing, vessel density **(A)**, diameter **(B)**, vessel area **(C)**, tortuosity **(D)**, velocity **(E)**, FRI **(F)** in men with and without azoospermia. Data is displayed as mean ± standard deviation. p values are calculated by Student’s two-tailed t-test or Mann-Whitney U test for parametric and non-parametric data, respectively. N=11 (men with HH off treatment), N=10 (men with HH on TRT) and N=9 (men with HH on gonadotrophins) except for (A) where N=12 (men with HH off treatment)

FRI, flow related index; a.u, arbitrary units; HH, hypogonadotrophic hypogonadism; TRT, testosterone replacement therapy


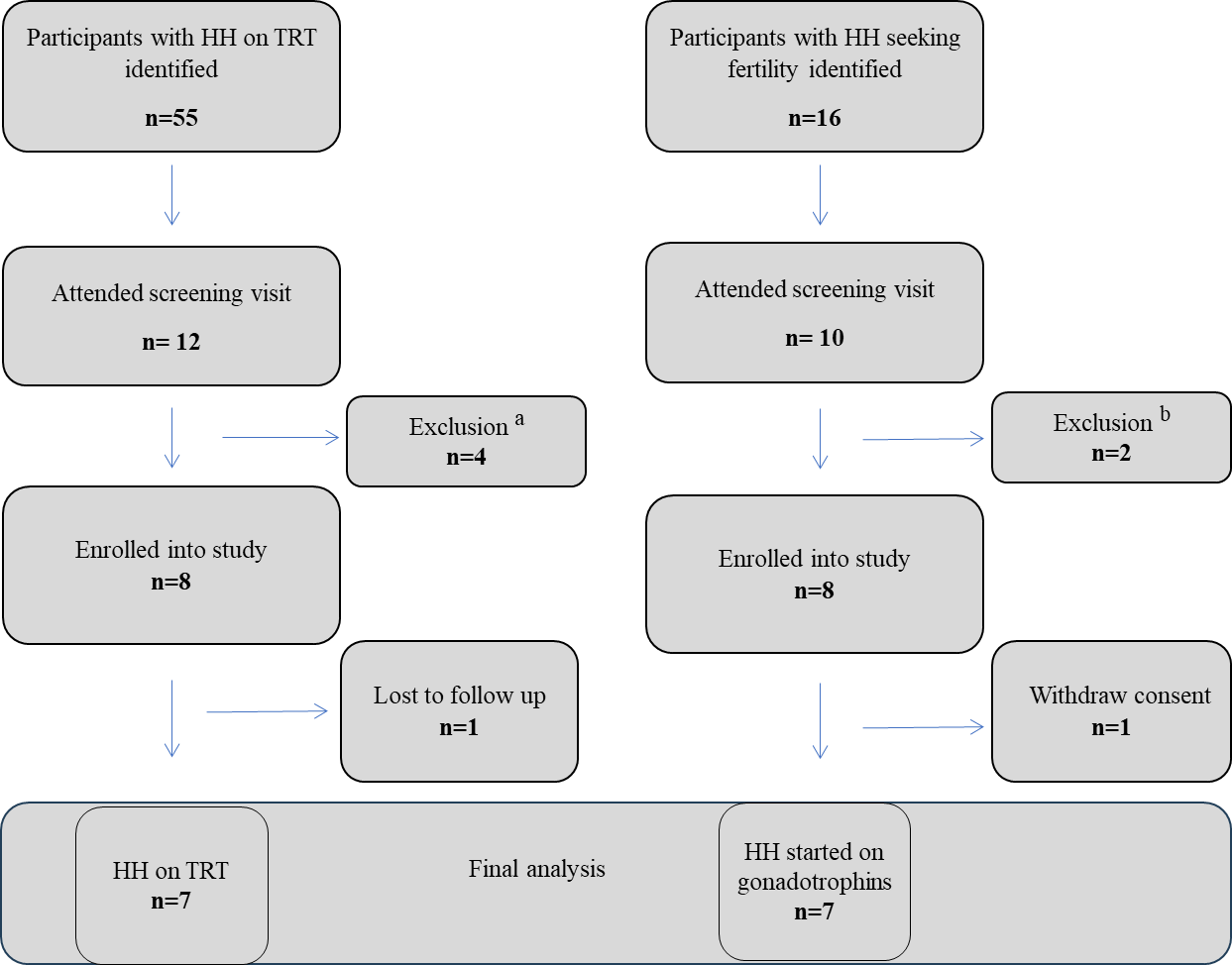


**Figure S11:** **Study 3 flow chart**

HH: hypogonadotrophic hypogonadism; TRT: testosterone replacement therapy

^a^ reason for exclusion: not interested

^b^ reason for exclusion: not diagnosed with HH

 
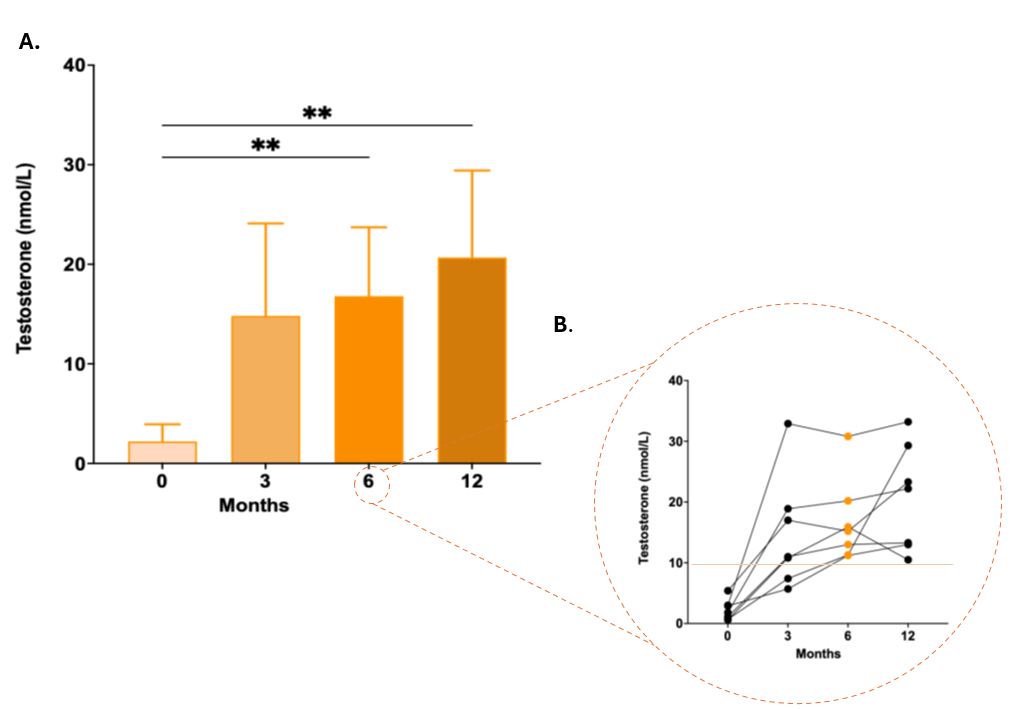


**Figure S12. Effects of gonadotrophins on serum testosterone levels in men with hypogonadotrophic hypogonadism
(A)** Bar graphs of mean serum testosterone at baseline, 3 months, 6 months and 12 months in men

receiving gonadotrophins hormone stimulation. Data are shown as means ± standard deviation.

**(B)** Plots of each individual participant plotted as mean ± standard deviation on the y-axis, over the four time points on the x-axis. The orange dashed line demonstrates serum testosterone within the normal range in all men by 6 months. **, p<0·01, by one-way ANOVA followed by Bonferroni’s post hoc analysis

   
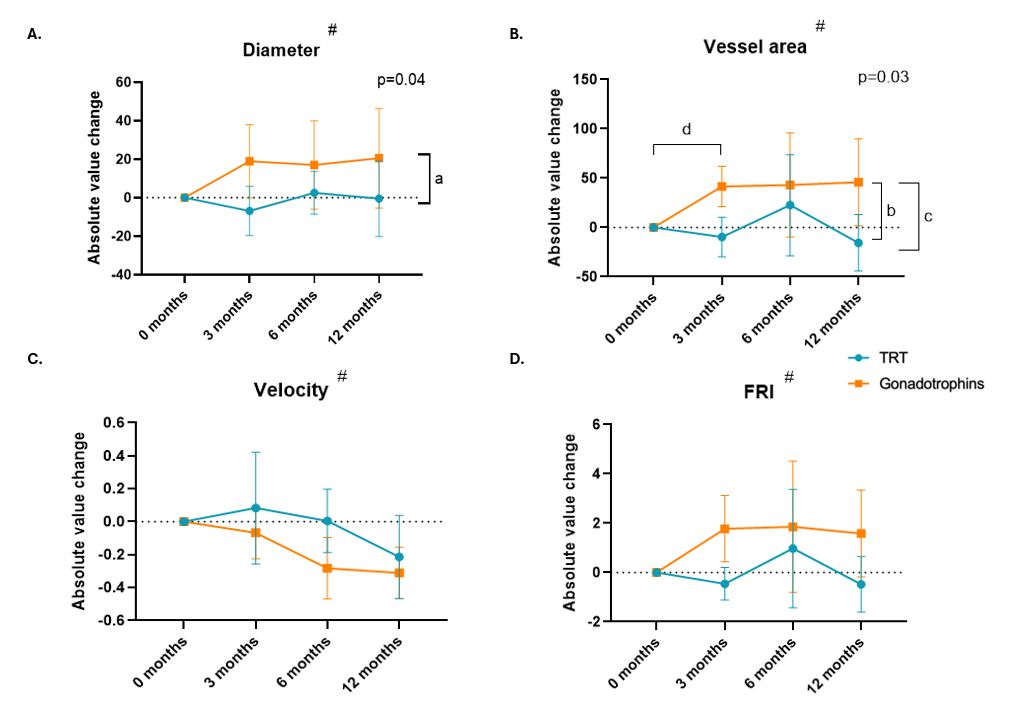


**Figure S13. Comparing changes in microvascular characteristics during gonadotrophins versus testosterone therapy in men with hypogonadotrophic hypogonadism**

**(A-D)** Time profiles of change of select microvascular parameters in men with HH on TRT and men with HH on gonadotrophins. Vessel diameter **(A)**, vessel area **(B)**, velocity **(C)**, FRI **(D)** plotted as mean ± standard deviation on the y-axis, over the four time points on the x-axis.

Inset is the results of the analysis of group by time interaction by mixed effects model

^#^ missing data (n=7, TRT; n=6, Gonadotrophins)

FRI, flow related index; TRT, testosterone replacement therapy

Post hoc analysis with Bonferroni’s multiple comparisons test

a: TRT vs. Gonadotrophins at 3 months, p=0·02; b: TRT vs. Gonadotrophins at 3 months, p<0·001; c: TRT vs. Gonadotrophins at 12 months, p=0·01; d: Gonadotrophins, 0 months vs.3 months, p=0·03.

  
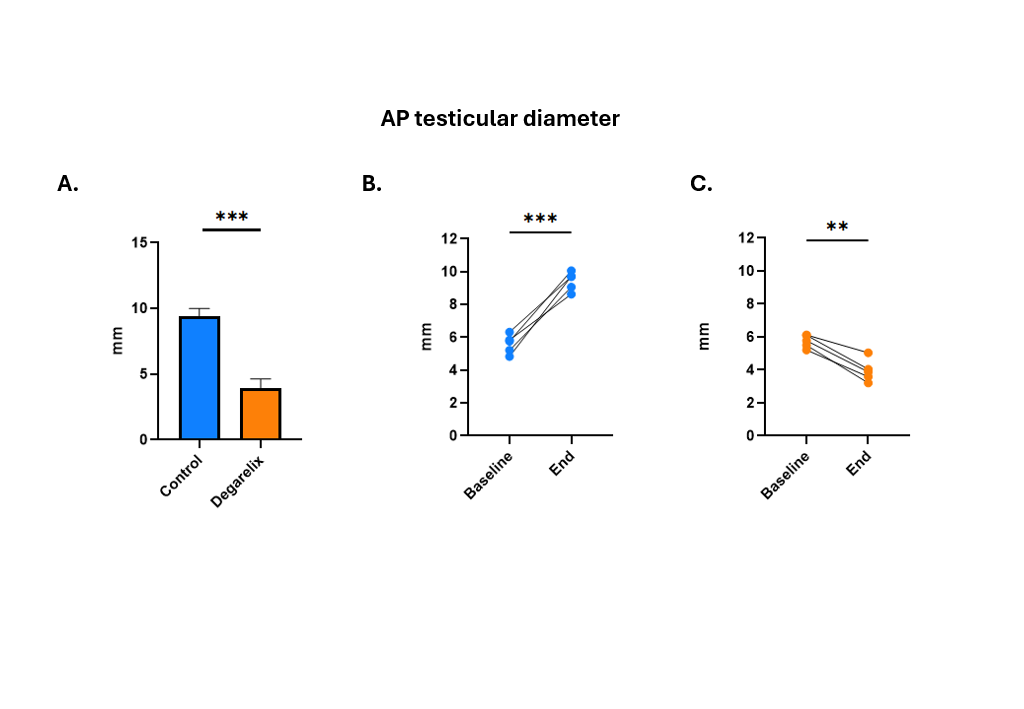


**Figure S14. Effect of pubertal blockade on testes development**

Comparisons of anteroposterior (AP) testicular diameter of (**A**) Degarelix injected rodent vs vehicle injected control at the end of the experiment (**B**) before and after treatment with vehicle injection, (**C**) before and after treatment with Degarelix.

** p<0·01. *** p<0·001, by Student’s two-tailed t-test. N=5 (Degarelix-injected rodents) and N=5 (vehicle-control injected rodents)

 
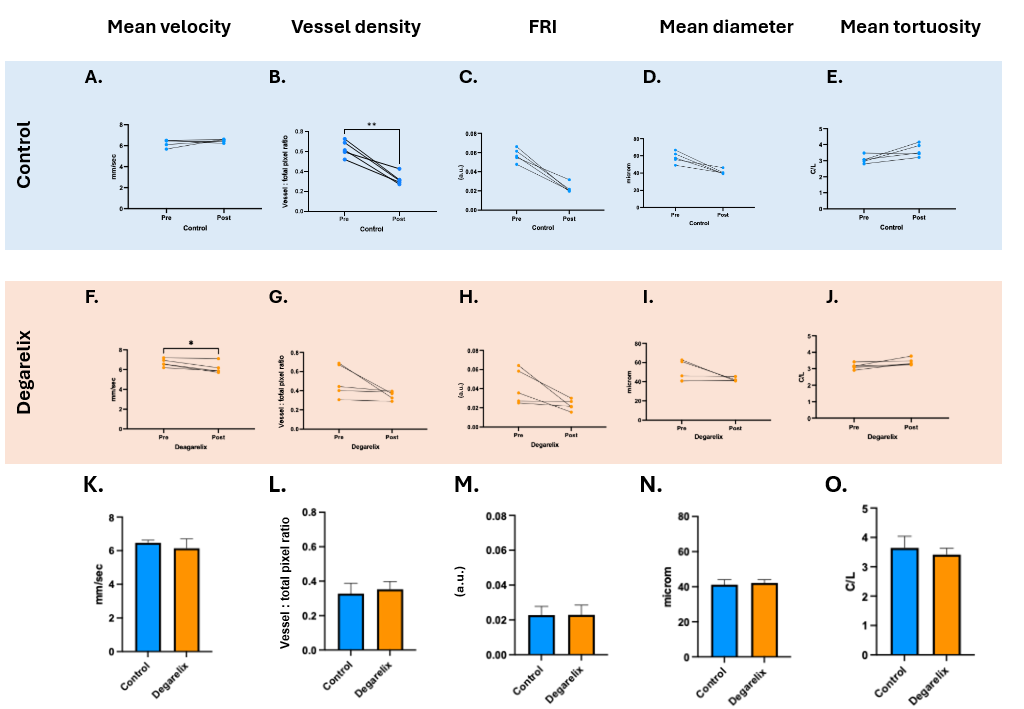


**Figure S15. Effect of pubertal blockade on microvascular development in rats**

**(A-E)** Comparisons of select testicular microvascular parameters of rodents before and after treatment with vehicle injection: Mean velocity **(A)**, vessel density **(B)**, FRI **(C)**, diameter **(D)**, tortuosity **(E)**. Level of significance is shown on the graph.

**(F-J)** Comparisons of select testicular microvascular parameters of rodents before and after treatment with Degarelix: Mean velocity **(F)**, vessel density **(G)**, FRI **(H)**, diameter **(I)**, tortuosity **(J)**. Level of significance is shown on the graph.

**(K-O)** Comparison of testicular vessel area of Degarelix injected rodent vs vehicle injected control. Mean velocity **(K)**, vessel density **(L)**, FRI **(M)**, diameter **(N)**, tortuosity **(O).** Data is displayed as mean ± standard deviation

* p<0·05, ** p<0·01, by paired t test. N=5 (Degarelix-injected rodents) and N=5 (vehicle-control injected rodents)

FRI, flow related index; a.u, arbitrary units

 
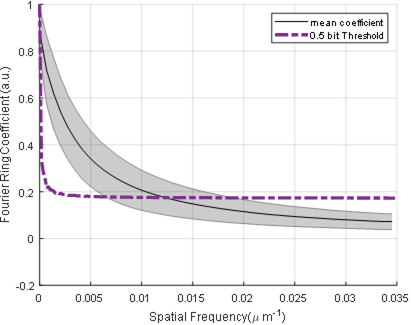


**Figure S16 Estimated imaging resolution using Fourier ring coefficient**

The mean coefficient curve is calculated from all super resolution imaging in this study. The shaded area indicates the standard deviation of the coefficients. The dashed line indicates the 0·5 bit threshold used for determining the cutoff spatial frequency. The estimated resolution is 1/(0·011 μm ^-1^) ≈ 90 μm.

     
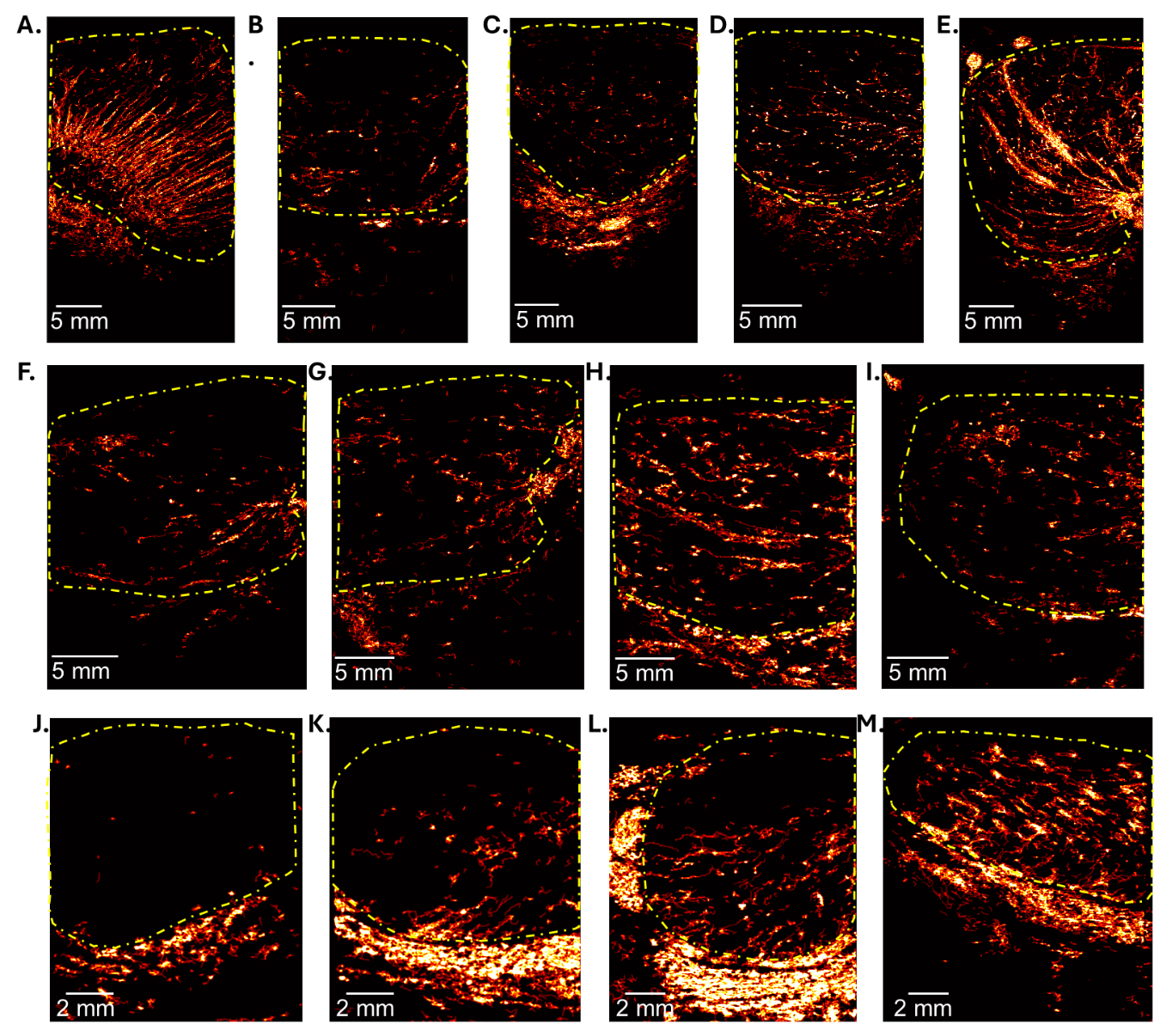


**Figure S17. Super resolution images of the testes in the transverse plane**.

Control (**A**) and man with HH (**B**) from study 1. HH off treatment (**C**), HH on TRT (**D**) and HH on gonadotrophins (**E**) from study 2. Participant on testosterone replacement therapy at baseline/0 month (**F**), 3 months (**G**), 6 months (**H**), and 12 months (**I)** and participant on gonadotrophins at baseline/0 month (**J**), 3 months (**K**), 6 months (**L**), and 12 months into treatment (**M**) from study 3.

The intratesticular region of interest is marked with the yellow dotted line.

**Supplemental tables:**

Abbreviations used:

BMI, Body Mass Index

ED, erectile dysfunction

ETOH, alcohol

HH, hypogonadotrophic hypogonadism

ISCO-08, international standard of classification of occupations

TRT, testosterone replacement therapy.

* Denotes where differences between groups were statistically significant at p < 0·05.

**Table S1: Baseline demographics and anthropometrics of men with hypogonadotrophic hypogonadism versus controls**

Continuous data are presented as mean ± standard deviation, unless otherwise stated.

| **Baseline characteristic (units)** |  | **HH**  **n=9** | **Controls**  **n=10** | **p value** |
| --- | --- | --- | --- | --- |
| **Ethnicity** |  |  |  | 0.50 |
| *Caucasian* |  | 5 | 6 |  |
| *Asian* |  | 1 | 3 |  |
| *Black* |  | 0 | 0 |  |
| *Arab* |  | 1 | 0 |  |
| *Mixed* |  | 2 | 1 |  |
| **Occupation (Based on ISCO-08 classification)**  *Managers*  *Professionals*  *Technicians and Associate professionals*  *Clerical support workers*  *Service and sales workers*  *Unemployed* | |  |  | 0.50 |
|  |  | 1  5  0  0  1  2 | 0  6  0  0  3  1 |  |
| **Smoking status**  *Yes*  *No*  *Ex-smoker* |  | 1  6  2 | 0  7  3 | 0.54 |
| **ETOH current**  *Yes*  *No* |  | 5  4 | 6  4 | 0.66 |
| **History of infertility**  *Yes*  *No* |  | 3  6 | 0  10 | 0.05* |
| **Children**  *Yes*  *No* |  | 4  5 | 4  6 | >0.99 |
| **ED**  *Yes*  *No* |  | 4  5 | 0  10 | 0.03* |
| **Hypertension**  *Yes*  *No* |  | 1  8 | 0  10 | 0.47 |
| **Diabetes**  *Yes*  *No* |  | 0  9 | 0  10 | >0.99 |
| **Systolic blood pressure (mmHg)** |  | 130 ± 11 | 127 ± 11 | 0.57 |
| **Diastolic blood pressure (mmHg)** |  | 75 ± 13 | 83 ± 10 | 0.17 |

**Table S2: Baseline demographics and anthropometrics of men with hypogonadotrophic hypogonadism following gonadotrophin versus testosterone treatment.** Continuous data presented as mean ± standard deviation.

| **Baseline characteristic (units)** |  | **HH on gonadotrophins n=9** | **HH off-treatment**  **n=12** | **HH on TRT**  **n=11** | **p value** |
| --- | --- | --- | --- | --- | --- |
| **Ethnicity** |  |  |  |  | 0.64 |
| *Caucasian* |  | 6 | 6 | 6 |  |
| *Asian* |  | 0 | 1 | 0 |  |
| *Black* |  | 0 | 0 | 1 |  |
| *Arab* |  | 1 | 2 | 0 |  |
| *Mixed* |  | 2 | 3 | 4 |  |
| **Occupation (Based on ISCO-08 classification)**  *Managers*  *Professionals*  *Technicians and Associate professionals*  *Clerical support workers*  *Service and sales workers*  *Unemployed* | |  |  |  | 0.56 |
|  |  | 1  4  1  0  3  0 | 2  4  2  0  3  1 | 4  4  0  0  1  2` |  |
| **Smoking status**  *Yes*  *No*  *Ex-smoker* |  | 1  5  3 | 2  8  2 | 1  7  3 | 0.99 |
| **ETOH consumption**  *Yes*  *No* |  | 3  6 | 9  3 | 7  4 | 0.15 |
| **History of infertility**  *Yes*  *No* |  | 7  2 | 11  1 | 5  6 | 0.04* |
| **Children**  *Yes*  *No* |  | 1  8 | 3  9 | 7  4 | 0.03* |
| **ED**  *Yes*  *No* |  | 1  8 | 7  5 | 2  9 | 0.04* |
| **Onset of HH**  *Pre-pubertal onset*  *Post-pubertal onset* |  | 3  6 | 5  7 | 4  7 | 0.92 |
| **Hypertension**  ***Yes***  ***No*** |  | 0  9 | 2  10 | 2  9 | 0.40 |
| **Diabetes Mellitus**  *Yes*  *No* |  | 0  9 | 2  10 | 0  11 | 0.17 |
| **Systolic blood pressure (mmHg)** |  | 133 ± 11 | 128 ± 9 | 131 ± 15 | 0.72 |
| **Diastolic blood pressure (mmHg)** |  | 83 ± 6 | 83 ± 8 | 78 ± 14 | 0.50 |

**Table S3: Baseline demographics and anthropometrics of men with hypogonadotrophic hypogonadism starting gonadotrophins versus testosterone replacement therapy.** Continuous data presented as mean ± standard deviation.

| **Baseline characteristic (units)** |  | **Gonadotrophins**  **n=7** | **TRT**  **n=7** |  | **p value** |
| --- | --- | --- | --- | --- | --- |
| **Ethnicity** |  |  |  |  | 0.48 |
| *Caucasian* |  | 2 | 4 |  |  |
| *Asian* |  | 1 | 0 |  |  |
| *Black* |  | 0 | 0 |  |  |
| *Arab* |  | 2 | 0 |  |  |
| *Mixed* |  | 2 | 3 |  |  |
| **Occupation (Based on ISCO-08 classification)**  *Managers*  *Professionals*  *Technicians and Associate professionals*  *Clerical support workers*  *Service and sales workers*  *Unemployed* | |  |  |  | >0.99 |
|  |  | 2  2  1  0  1  1 | 3  2  0  0  1  1 |  |  |
| **Smoking status**  *Yes*  *No*  *Ex-smoker* |  | 1  4  2 | 0  5  2 |  | >0.99 |
| **ETOH consumption**  *Yes*  *No* |  | 5  2 | 5  3 |  | >0.99 |
| **History of infertility**  *Yes*  *No* |  | 7  0 | 3  4 |  | 0.07 |
| **Children**  *Yes*  *No* |  | 3  4 | 5  2 |  | 0.60 |
| **ED**  *Yes*  *No* |  | 3  4 | 0  7 |  | 0.19 |
| **Onset of HH**  *Pre-pubertal onset*  *Post-pubertal onset* |  | 4  3 | 2  5 |  | 0.59 |
| **Hypertension**  *Yes*  *No* |  | 2  5 | 1  6 |  | >0.99 |
| **Diabetes Mellitus**  *Yes*  *No* |  | 0  7 | 1  6 |  | >0.99 |
| **Systolic blood pressure (mmHg)** |  | 131 ± 8 | 130 ± 17 |  | 0.86 |
| **Diastolic blood pressure (mmHg)** |  | 84 ± 9 | 78 ± 12 |  | 0.33 |

**Table S4. Semen analysis results of the 7 participants receiving gonadotrophins therapy, before (0 months) and 12 months into treatment.**

| **Participant** | **0 month** | **12 months of gonadotrophins therapy** |
| --- | --- | --- |
| **Participant 1** | Sperm concentration:0  Semen volume: 1·5 ml | Sperm concentration:0  Semen volume: 3·5 ml |
| **Participant 2** | Sperm concentration:0  Semen volume: 6·5 ml | Sperm concentration:0·3  Semen volume: 3·2 ml  Total motility:62%  Progressive motility: 60%  Total motile count: 0.95 |
| **Participant 3** | Sperm concentration:43  Semen volume: 1·9 ml  Total motility:12%  Progressive motility: 7%  Total motile count: 9·8 | Sperm concentration:18.3  Semen volume: 6·1 ml  Total motility:52%  Progressive motility: 47%  Total motile count: 62 |
| **Participant 4** | Sperm concentration:0  Semen volume: 1·3 ml | Sperm concentration:0  Semen volume: 0·8 ml |
| **Participant 5** | Sperm concentration: 0  Semen volume: 2·3 ml | Sperm concentration:0  Semen volume: 2·7 ml |
| **Participant 6** | Sperm concentration:2·3  Semen volume: 1 ml  Motile and immotile sperm seen  Total motile count: NA | Sperm concentration:80  Semen volume: 2·2 ml  Total motility:63%  Progressive motility: 62%  Total motile count: 111 |
| **Participant 7** | Sperm concentration: 0  Semen volume: 0·9 ml | Sperm concentration:0·1  Semen volume: 0·7 ml  Motile and immotile sperm seen |

**Table S5: Baseline microvascular parameters of rodents.** Continuous data are presented as mean ± standard deviation (95% CI) unless otherwise stated.

| Baseline characteristics (units): | Degarelix group (n=5) | Control group (n=5) | p value |
| --- | --- | --- | --- |
| Vessel area (mm^2^) | 41·71 ± 8·798 (30·79-52·64) | 40·80 ± 5·98 (33·38-48·23) | 0·853 |
| FRI (a.u) | 0·042 ± 0·018 (0·019-0·064) | 0·0572 ± 0·007 (0·049-0·066) | 0·118 |
| Vessel density (Ψ) | 0·50 ± 0·17 (0·29-0·71) | 0·63 ± 0·08 (0·53-0·73) | 0·157 |
| Vessel diameter (microns) | 50·27 ± 10·76 (36·91-63·64) | 58·21 ± 6·68 (49·93-66·50) | 0·309 |
| Mean velocity (mm/sec) | 6·69 ± 0·39 (6·21-7·17) | 6·26 ± 0·36 (5·81-6·71) | 0·110 |
| Mean tortuosity (C/L) | 3·15 ± 0·18 (2·92-3·73) | 3·07 ± 0·25 (2·77-3·38) | 0·615 |

Ψ Vessel density has no unit. It is a ratio of number of vessels : number of pixel

FRI, flow related index; a.u, arbitrary units

**Table S6** **Total body weight, testes weight and anteroposterior testicular diameter for all rodents at the start and end of experiment**.

| Group allocation | Start of the experiment | | End of the experiment | |
| --- | --- | --- | --- | --- |
| Control 1 | Total body weight (gr) | 83 | Total body weight (gr) | 329 |
|  | Testes weight (gr) | na | Testes weight (gr) | 1.56 |
|  | AP testicular diameter (mm) | 6.32 | AP testicular diameter (mm) | 9.70 |
| Control 2 | Total body weight (gr) | 84 | Total body weight (gr) | 312 |
|  | Testes weight (gr) | Na | Testes weight (gr) | 1.58 |
|  | AP testicular diameter (mm) | 5.84 | AP testicular diameter (mm) | 8.63 |
| Control 3 | Total body weight (gr) | 70 | Total body weight (gr) | 310 |
|  | Testes weight (gr) | Na | Testes weight (gr) | 1.63 |
|  | AP testicular diameter (mm) | 5.76 | AP testicular diameter (mm) | 10.06 |
| Control 4 | Total body weight (gr) | 84 | Total body weight (gr) | 359 |
|  | Testes weight (gr) | Na | Testes weight (gr) | 1.64 |
|  | AP testicular diameter (mm) | 5.21 | AP testicular diameter (mm) | 9.06 |
| Control 5 | Total body weight (gr) | 77 | Total body weight (gr) | 344 |
|  | Testes weight (gr) | Na | Testes weight (gr) | 1.52 |
|  | AP testicular diameter (mm) | 4.83 | AP testicular diameter (mm) | 9.72 |
|  | | | | |
| Degarelix 1 | Total body weight (gr) | 87 | Total body weight (gr) | 284 |
|  | Testes weight (gr) | na | Testes weight (gr) | 0.09 |
|  | AP testicular diameter (mm) | 5.50 | AP testicular diameter (mm) | 3.21 |
| Degarelix 2 | Total body weight (gr) | 93 | Total body weight (gr) | 284 |
|  | Testes weight (gr) | Na | Testes weight (gr) | 0.12 |
|  | AP testicular diameter (mm) | 6.12 | AP testicular diameter (mm) | 5.04 |
| Degarelix 3 | Total body weight (gr) | 81 | Total body weight (gr) | 297 |
|  | Testes weight (gr) | Na | Testes weight (gr) | 0.09 |
|  | AP testicular diameter (mm) | 5.78 | AP testicular diameter (mm) | 3.88 |
| Degarelix 4 | Total body weight (gr) | 77 | Total body weight (gr) | 266 |
|  | Testes weight (gr) | Na | Testes weight (gr) | 0.09 |
|  | AP testicular diameter (mm) | 6.10 | AP testicular diameter (mm) | 4.04 |
| Degarelix 5 | Total body weight (gr) | 72 | Total body weight (gr) | 270 |
|  | Testes weight (gr) | Na | Testes weight (gr) | 0.09 |
|  | AP testicular diameter (mm) | 5.21 | AP testicular diameter (mm) | 3.59 |

Supplementary videos

Video S1: MB tracking and microvasculature reconstruction video corresponding to Figure 2A. The left panel video presents the CEUS videos after noise thresholding; the red trajectories indicate the MB tracking. On the right panel, the accumulated MB density map over time was presented.

Video S2: MB tracking and microvasculature reconstruction video corresponding to Figure 2B. The left panel video presents the CEUS videos after noise thresholding; the red trajectories indicate the MB tracking. On the right panel, the accumulated MB density map over time was presented.
